# Seismic Stereometry Reveals Preparatory Behavior and Source Kinematics of Intermediate‐Size Earthquakes

**DOI:** 10.1029/2020GL088563

**Published:** 2020-09-02

**Authors:** A. Mordret, F. Brenguier, M. Causse, P. Boué, C. Voisin, I. Dumont, F. L. Vernon, J. P. Ampuero

**Affiliations:** ^1^ Université Grenoble Alpes, Université Savoie Mont Blanc, CNRS, IRD, IFSTTAR, ISTerre Grenoble France; ^2^ TOTAL CSTJF Pau France; ^3^ Scripps Institution of Oceanography University of California, San Diego La Jolla CA U.S.A; ^4^ Université Côte d'Azur, IRD, Observatoire de la Côte d'Azur, CNRS, Géoazur Valbonne France

## Abstract

Although moderate‐size earthquakes are poorly studied by lack of near‐fault observations, they can provide key information about larger damaging earthquakes. Here we propose a new approach, inspired by double‐difference relocation, that uses high‐coherency waveforms recorded at neighboring sensors, to study the preparation phase and dynamics of moderate‐size earthquakes. We validate this technique by analyzing the 2016, *M*_*w*_5.2 Borrego Springs earthquake in Southern California and find consistent rupture velocities of 2 km/s highlighting two main rupture asperities. The analysis of the 2019, *M*l5.2 Le Teil earthquake in France reveals slow nucleation at depth that migrates to the surface and propagates northward with a velocity of ∼2.8 km/s, highlighting two main rupture events also imaged by InSAR. By providing unprecedented resolution in our observation of the rupture dynamics, this approach will be useful in better understanding the preparation phase and rupture of both tectonic and induced earthquakes.

## Key Points

- Stereometry uses stereoscopic effect to study intermediate‐size earthquake sources
- Stereometry retrieves finite fault rupture parameters such as direction, length, and speed
- Stereometry can reveal the activity of a single fault patch before an earthquake

## 1 Introduction

Questions such as what drives small rupture instability (Campillo & Ionescu, 1997; Dieterich, 1979; Latour et al., 2013; Rubin & Ampuero, 2005) to grow seemingly to either small or large earthquakes, or what a fault rupture history can tell about its potential to generate a large earthquake (Bouchon et al., 2011, 2013; Ellsworth & Bulut, 2018; Ruiz et al., 2017; Tape et al., 2018; Yoon et al., 2019), are still largely debated in seismology (Ellsworth & Beroza, 1995; Beroza & Ellsworth, 1996; Mori & Kanamori, 1996; Mignan, 2014; Olson & Allen, 2005). Because they occur often and widely, a more systematic analysis of the source of small to moderate‐size earthquakes (*M*_*w*_ <∼5.5) could unravel crucial information about the mechanisms of earthquakes sources. Because of their relatively small size and the lack of near‐fault observations, the preparation and nucleation phases of small and moderate‐size earthquakes are poorly understood, and compared to large earthquakes, their whole rupture history is rarely studied comprehensively. In this paper, we propose an approach to study earthquake rupture based on highly coherent seismic waveforms recorded at two neighboring stations.

While the location and time are basic information available in catalogs even for small earthquakes, it is often based on a point source approximation. The main reason is the lack of independent data (near and far‐field seismic, geodetic) needed to constrain the complexity of a finite fault rupture (McGuire, 2004). When available, their analyses show that small earthquake ruptures can be as complex as large earthquake ones (Boatwright, 2007; Courboulex et al., 2013; Fan & McGuire, 2018; Ross et al., 2017), with varying source duration, rupture velocity, directivity or nonuniform slip distribution along a finite fault. Assessing these attributes accurately for small‐to‐intermediate earthquakes is of paramount importance to constrain fundamental earthquake physics and scaling laws across all magnitude ranges, such as the approximately constant stress‐drop for all earthquake sizes (Aki, 1967; Shaw, 2009). Uncertainties in these scaling laws are one of the largest sources of uncertainties in earthquake hazard assessment (Nievas et al., 2020). Better characterizing finite fault rupture kinematics, such as directivity has also large implications for seismic hazard estimation because it tends to amplify the ground motion in the direction of rupture, even for small earthquakes (Bakun et al., 1978; Ben‐Menahem, 1961, 1962; Boatwright & Boore, 1982; Boatwright, 2007; Boore & Joyner, 1978; Courboulex et al., 2013). Estimating these parameters is done routinely for large earthquakes by inverting seismic and geodetic data (Delouis et al., 2002). These methods rely on the forward modeling of the data using an estimate of the Green's function between the discrete sources and the observation points. For teleseismic data of large earthquakes, global velocity models are used to compute the Green's functions (Vallée et al., 2011). For strong motion and regional seismic data, the Green's function is either calculated from local velocity models (Hartzell & Heaton, 1983) or assumed empirically from a small aftershock or foreshock (Causse et al., 2017; Hartzell, 1978; McGuire, 2004; H.  Meng et al., 2020). In both cases, the uncertainties on the structure or the small event induce uncertainties on the source parameters of the mainshock (Mai et al., 2016).

Another way to estimate some finite fault earthquake source properties is by using a dense seismic network with the back‐projection technique which maps the spatiotemporal distribution of high‐frequency radiations along the fault (Bao et al., 2019; Ishii et al., 2005; Kiser & Ishii, 2017; L.  Meng et al., 2011; Xu et al., 2009). While this latter technique does not require a proper inversion nor an accurate velocity model for teleseismic distances, it needs dense enough seismic arrays for the wavelength of analysis. Current permanent dense arrays have interstation distances that make the back projection only suitable for large earthquakes. The recent emergence of large N, very dense arrays of nodal geophones (Ben‐Zion et al., 2015; Brenguier et al., 2016, 2019; Dougherty et al., 2019; Fan & McGuire, 2018; Hansen & Schmandt, 2015; Li et al., 2018) has the potential to make the study of smaller magnitude earthquakes by back‐projection possible, but their short‐term deployment hinders their use for long‐term, systematic earthquake source analysis. Distributed acoustic sensing on fiber optic cables is paving the way toward hyperdense permanent seismic arrays (Jousset et al., 2018), even on the seafloor (Sladen et al., 2019). To improve our understanding of fault rupture, we need to gain more information from seismic sensors located mostly at the Earth's surface.

In this study, we present a new data‐driven method, called seismic stereometry, to estimate finite fault rupture parameters for intermediate‐size earthquakes, which does not rely on inversion nor deconvolution by an empirical or synthetic Green's function. Similarly to back‐projection, our method analyzes the high‐frequency *P* waves emitted by the source. However, we relax the need of having a dense network (Abercrombie & Mori, 1994; Hough, 1994; Hough et al., 1993; Fletcher et al., 2006; Ishii et al., 2005; L.  Meng et al., 2011, 2014) by studying seismograms recorded at only two neighboring stations. In the following sections, we describe the conditions of application of this technique and show applications on two recent earthquakes: the *M*_*w*_ 5.2, 2016 Borrego Springs earthquake (Ross et al., 2017), and the *M*_*Lv*_ 5.2, 2019 Le Teil earthquake in France. This simple technique can help improve the statistics on finite fault rupture parameters for intermediate‐size earthquakes and can be used to study the preparation phase and foreshock activity before the main events. Because of the targeted earthquake size, we believe that seismic stereometry can have a strong potential to study, among others, induced seismicity generated by resource production and help mitigate the associated seismic risk.

## 2 Seismic Stereometry

Seismic stereometry relies on the elevated coherency of the high‐frequency (higher than the corner frequency) component of the source function (Heaton, 1990; Madariaga, 1977; Ruiz et al., 2011) observed at two neighboring stations to estimate, using fine differential traveltime measurements, the spatial evolution of the rupture along the fault. Given the proper geometry between the fault and the pair of stations, the dynamics of the rupture creates a stereoscopic effect with the two seismograms. Quantifying this effect gives clues about the rupture length, the rupture velocity, and the rupture direction. The following derivations of the stereometric equations are only valid for a horizontally propagating rupture. Along‐dip propagation could in theory be analyzed but the trade‐off between the horizontal propagation and along‐dip propagation cannot be solved with only two stations at the surface. Extra stations would be needed. A more general framework involving along‐dip propagation and an inhomogeneous and anisotropic velocity model requires a careful derivation of the second‐order approximation of the traveltime between the events and the receivers with respect to the events and receivers positions. This work is beyond the scope of the present paper and will be the subject of a subsequent contribution.

**Figure 1:**
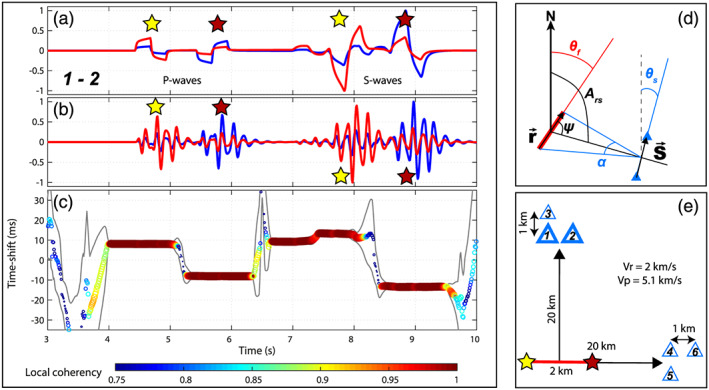
Stereometry principles: synthetic cases. (a) Raw seismograms for station Pair 1–2. The blue trace is from the first station, the red trace is from the second station. The stars denote the phases generated by the start and the end of the rupture. (b) Same seismograms as (a) filtered between 5 and 12 Hz. (c) Stereometry measurements: the colored circles show the local time shift between the two traces and their local coherency. The gray contours show the confidence interval of the time shift measurements. (d) Definition of the parameters used to determine *L*_*r*_ and *V*_*r*_. (e) Sketch of the modeling setup.

Following a double‐difference approach (Got et al., 1994; Waldhauser & Ellsworth, 2000), assuming that the epicentral distance *D* is much larger than the two stations separation and much larger than the rupture length, we can estimate the rupture length *L*_*r*_ and the rupture velocity *V*_*r*_ from the seismograms. By measuring the time difference $\Delta t_{r}^{s_{1}}$ and $\Delta t_{r}^{s_{2}}$ between the first and the last high‐frequency pulse emitted by the source at station *s*_1_ and *s*_2_ and the arrival time difference of each pulse at the two stations, $\Delta t_{s_{1}s_{2}}^{lastpulse}-\Delta t_{s_{1}s_{2}}^{firstpulse}=\Delta \delta t$, we obtain:

(1) $$L_{r}≃\frac{D|\alpha |}{\sin(\psi )}=\frac{D|\Delta \delta t|}{p\sin(\psi )(\delta x_{s}\cos A_{rs}-\delta y_{s}\sin A_{rs})}\;,$$

where *p* is the ray parameter between the rupturing point and the barycenter of the station pair, *α* the angle under which the two stations see the rupture length, *A*_*rs*_, the azimuth from the rupture to the stations, *ψ* the angle between the strike of the rupture and the stations, and **S** = (*δx*_*s*_, *δy*_*s*_, *δz*_*s*_) the vector linking the pair of stations (Figure 1d). The sign of Δ*δt* gives the direction of propagation: Δ*δt* > 0 means that the rupture is getting closer to station *s*_1_ or away from station *s*_2_. If the rupture propagates mostly horizontally, we have |**r**|=(*δx*_*r*_, *δy*_*r*_, *δz*_*r*_ ≃ 0) ≃ *L*_*r*_ the vector linking the hypocenter to the end of the rupture so that the rupture velocity is obtained with

(2) $$V_{r}=\frac{\left|r\right|}{\delta \tau }≃\frac{2L_{r}V_{h}}{V_{h}(\Delta t_{r}^{s_{1}}+\Delta t_{r}^{s_{2}})+2L_{r}\cos(\psi )}\;,$$

where *δτ* is the true rupture duration and *V*_*h*_ the velocity in the hypocenter region, assumed constant (Figure 1d). Details of the derivation of equations 1 and 2 are given in the supporting information Text S1.

By definition, stereometry can only be applied if Δ*δt* ≠ 0 to observe a stereoscopic effect. This condition imposes that *α* ≠ 0 or equivalently *ψ* ≠ 0, that is, if the barycenter of the two stations is not aligned with the fault. Equation (1) dictates other geometrical constraints on the rupture/receiver‐pair geometry so that the denominator is nonzero. The condition $p\sin(\psi )(\delta x_{s}\cos A_{rs}-\delta y_{s}\sin A_{rs})\neq 0$ means that the earthquake cannot be at the vertical of the stations or that the earthquake and the two stations are not colinear (*θ*_*s*_ ≠ *A*_*rs*_, *θ*_*s*_ being the azimuth from *s*_1_ to *s*_2_). From Equation 2, we can see that having *ψ* ≃ 90° alleviates the need to know the velocity structure around the hypocenter to get an accurate rupture velocity. In this case, a good approximation of the rupture velocity can be $V_{r}\approx L_{r}/\Delta t_{r}^{s_{1}}$. By principle, stereometry relative time shifts (Δ*δt*) are second‐order perturbations of the traveltime along a ray resulting from perturbations of its two endpoints. Therefore, as for double‐difference relocation, the heterogeneities along the ray are canceled and only perturbations on the source side and the receiver side are kept, minimizing the effects of lateral heterogeneities on the results.

We illustrate the principles of seismic stereometry with simple synthetic cases illustrated in Figure 1. We simulate an earthquake rupturing a 2 km long fault with a rupture velocity of 2 km/s in a homogeneous half‐space with *Vp* = 5.1 km/s and $Vs=Vp/\sqrt{3}$. The waveform modeling is done using the discrete wave number method of Bouchon and Aki (1977) implemented in the AXITRA software (Coutant, 1989). We used two point sources located at the beginning (yellow star in Figure 1e) and the end (dark red star in Figure 1e) of the fault that fire with 1 s delay. The point sources are triangle source functions with 0.5 s duration and right‐lateral strike‐slip motion. They are located at 12 km depth. Two groups of three stations are located 20 km from the earthquake, either in a direction perpendicular to the rupture or in the direction of the rupture. The stations are separated by 1 km and configured in an L‐shape geometry (Figure 1e). According to the geometrical constraints to apply stereometry, only station Pair 1‐2 is in the right configuration. The waveforms for this station pair are shown in Figures 1a and 1b).

Figure 1c shows the time delay seismic stereometry measurements. The instantaneous time shift between the two seismograms is measured using the cross‐wavelet transform method of Mao et al. (2019), using a Morlet wavelet. The cross‐wavelet transform method has the advantage to provide instantaneous (one value per data sample) and stable time shift estimations at several discrete frequencies at once. This alleviates the need to filter the data in different frequency bands and repeat the time shift measurement in each frequency band, as would be done with a moving‐window cross‐spectral analysis (Clarke et al., 2011; Poupinet et al., 1984). The instantaneous time shift between the wavelet transforms of the seismograms is measured for frequencies between 5 and 12 Hz. The displayed time shift is the average time shift in the 5–12 Hz frequency band, the time shift uncertainties are estimated as the time shift standard deviation in the same frequency band. The time shift between the two traces for the first *P* wave pulse is 8.1 ± 0.5 ms, the hypocenter being slightly closer to Station 1 than Station 2. This value is very close to the theoretical value of 8.4 ms. For the second *P* wave pulse, 1 s later, the time shift is −8.1 ± 0.5 ms. With these values, using Equations 1 and 2, the rupture length and rupture velocity are *L*_*r*_ = 1.927 ± 0.119 km and *L*_*r*_ = 1.927 ± 0.119 km/s, respectively. We also see that, in theory, stereometry can by applied on *S* wave pulses as well. However, in practice, the *S* waves are noisier and potentially polluted by the coda of the *P* wave which hampers an accurate measurement of Δ*δt*.

The high‐frequency pulses in the early seismograms are generated by acceleration and deceleration of the slip of each source and should be representative of the beginning and end of the slip of each source. These individual pulses should be observable at periods shorter than the acceleration and deceleration phases of each slip sequence (above several tens of Hz in frequency for small and intermediate‐size earthquakes). However, to keep high coherencies between the waveforms, we need to avoid frequencies too high for which scattering and attenuation dominate. Therefore, stereometry should be performed on the lower end of the high‐frequency component of the source where the temporal resolution is enough to separate broad pulses associated with each source but not enough to isolate the shorter pulses associated with the ramping up and down of the slip of each patch.

We show three other station configurations (Pairs 1‐3, 4‐5, and 4‐6) in the supporting information (Figures S1 to S3) which do not fulfill the geometrical requirements stated above, inducing cycle skipping for Pairs 1‐3 and 4‐6, with a decrease of the coherency between the seismograms, a larger uncertainty on the time shift measurements and an inaccurate absolute time shift estimation. For station Pair 4‐5, in the direction of the rupture, the seismograms are shorter in time because of the directivity effect. However, no relative time shift between the seismograms can be observed because the two stations are always equidistant to the source, for the whole rupture duration. The potential effects of the radiation pattern are discussed in the supporting information Text S2. To assess the potential effects of scattering and interference of reflected and refracted phases on the stereometry measurements, we performed the same synthetic tests in a more complex layered velocity model (Table S1) with varying hypocentral depths and rupture velocity (Figures S4 to S7). The results suggest that as long as the scattering is low enough and does not distort the waveform corresponding to the end of the rupture, the relative time shift between different stations can be measured, albeit with a larger uncertainty. However, it becomes more difficult to pick the traveltime difference between the last and first pulses, reducing, even more, the accuracy of the rupture velocity estimation.

## 3 Case Studies

### 3.1 The Borrego Springs Earthquake

**Figure 2:**
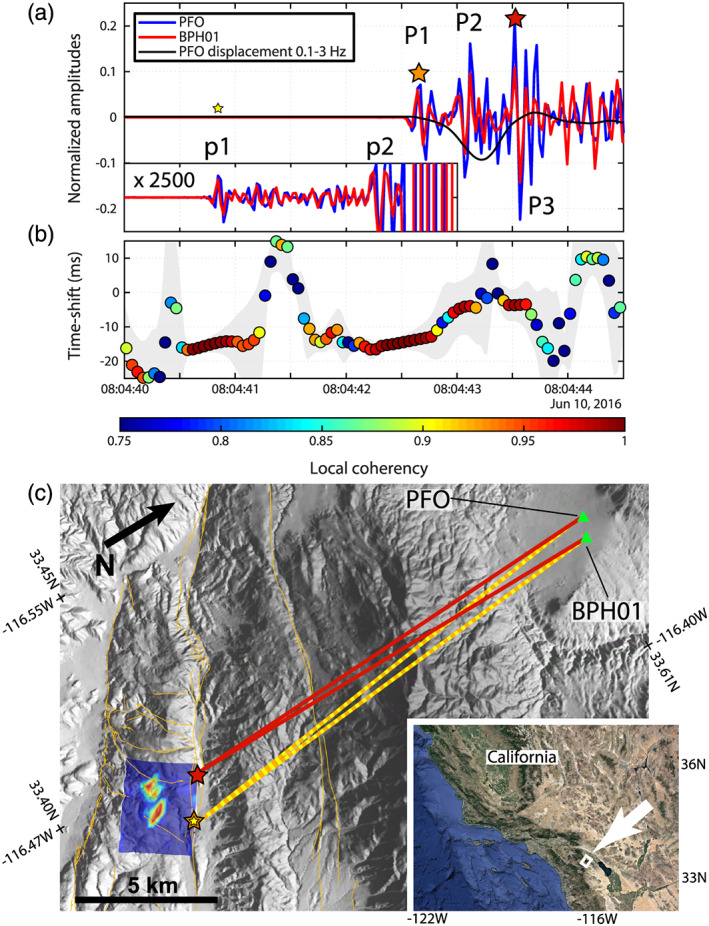
Stereometry application to the 2016 *M*_*w*_ 5.2 Borrego Springs earthquake. (a) Vertical velocity traces at stations PFO (blue) and BPH01 (red) filtered between 5 and 12 Hz without time shift correction. The long period displacement at PFO is shown in black. The inset shows a zoom on the trace before the mainshock highlighting a small precursory event. The stars show the different subevents of the seismic sequence. (b) Stereometry analysis. The gray area delineates the time shift confidence interval. (c) Map of the location of the Borrego Springs earthquake and the two stations used for the stereometry analysis. The blue and red image on the left shows the slip model of Ross et al. (2017) displayed horizontally on the side of the Clark Fault for interpretation of the stereometry results. The orange lines on the topographic background depict the regional seismogenic faults. The bottom right inset shows the location of the studied area.

To illustrate the use of seismic stereometry on real data we chose a well‐studied earthquake with strong directivity, surrounded by a dense network of seismic stations. The 2016, *M*_*w*_ 5.2 Borrego Springs earthquake (SCSN‐ID:37374687; 10 June 2016 08:04:39; latitude = 33.4441226°, longitude = −116.434491°, depth = 12.31 km) ruptured a 2 km long portion of the Clark Fault strand in the central part of the San Jacinto Fault Zone (SJFZ), Southern California (Figure 2). The rupture mechanism was mostly strike slip, the rupture starting from the southeast and propagating to the northwest with two main slip patches that reach 2 m displacement each (Ross et al., 2017). This rupture dynamics resulted in a strong directivity effect toward the northwest.

To resolve details along the 2 km rupture length, we need wavelengths smaller than 1 km, which means frequencies above 5.5 Hz, taking *Vp* = 5.5 km/s. Given the local *P* wave attenuation *Q*_*p*_= [300–800] (Hauksson & Shearer, 2006), the maximum distance range from the earthquake for which we can expect high‐quality data (with an amplitude reduction at the station of less than 50%) has to be smaller than 20–60 km at 10 Hz. Taking 10 Hz as the dominant frequency, we looked for pairs of stations for which the differential epicentral distance Δ*D* is related to the dominant wavelength by Δ*D* < *λ*/4 = 140 m, *λ*/4 = 140m < *Dist*(*sta*1, *sta*2) < 3*λ* = 1,650 m and epicentral distances smaller than 60 km. This distance filter provided us with 18 pairs of broadband stations, the additional azimuthal criteria removed one pair. The 17 remaining pairs of stations are all located at the Piñon Flat Observatory where high coherency of the seismic signals is to be expected (Vernon et al., 1991). The 13 stations involved in the 17 pairs are listed in Table S2 in the supporting information.

After visual inspection, most of the 17 pairs are suitable for stereometry analysis. We use the station pair PFO‐BPH01 to illustrate our measurements and show all the pairs in Figures S8–S24 in the supporting information (Text S3). The stations PFO and BPH01 exhibit a very high coherency for the first seconds of the *P* wave on the vertical components (Figure 2a). At high frequency, the coherency and the SNR are the highest between 5 and 12 Hz, the frequency band for which we applied stereometry (Figure 2b).

The high‐frequency *P* wave exhibits three main pulses (labeled P1, P2, and P3), P1 and P3 (orange and red stars in Figure 2a), corresponding to the starting and ending of the rupture, respectively, are separated by about *P*_*duration*_ = 0.925 s, in good agreement with Ross et al. (2017). If no other independent information is available, an estimate of the source duration can be obtained by the duration of the low‐frequency *P* wave displacement corresponding with high values of the local coherency. The stereometry analysis shows that the relative time shift varies for each pulse and increases from $\Delta t_{PFO-BPH01}^{P1}=-13.5$ ms for P1 to $\Delta t_{PFO-BPH01}^{P3}=-3.68$ ms for P3. Given the strike of the fault and the geometry of the two stations, this decrease of time shift indicates that the source was getting closer to PFO while the rupture was progressing (Figure 2c), in agreement with the reported SE‐NW rupture propagation (Ross et al., 2017).

Assuming *Vp* = 6.0 km/s at the hypocenter, with a departure angle of the ray to the stations about 90°, *θ*_*f*_ = 304°, an epicentral distance *D* = 18.67 km, an azimuth to the station pair *A*_*rs*_ = −10.36 ± 1.8° and *ψ* ≃50°, we find *L*_*r*_ = 3.37 ± 0.6 km and *V*_*r*_ = 2.6 km/s (∼76% *Vs*). Repeating this operation for all 17 pairs of stations and restricting the solutions to *V*_*r*_= [0.5–1.0]*Vs* and *L*_*r*_= [0.3–4.0] km, we find a mean rupture velocity of 1.98 ± 0.45 km/s (∼58 ± 13% *Vs*) and a mean rupture length of 2.3 ± 0.69 km. These values are in good agreement with the source model of Ross et al. (2017) although the rupture velocity might be underestimated with stereometry. The coordinates of the end of the rupture as well as its mean and uncertainty are shown in Figure S25 for the 12 pairs of stations for which we could estimate a rupture length and velocity reliably. The scatter in the estimated position comes from the uncertainties on Δ*δt* as well as the lateral heterogeneities of the crust in the vicinity of the sources and the stations, which are not taken into account in the method.

Beside key source parameters, stereometry analysis of the Borrego Springs earthquake can inform us about potential source processes. The time shift of the second *P* wave pulse (P2) is similar to the time shift of P3, indicating that they originate from the same area on the fault: The second asperity.P2 is likely the *P* wave signal originating from the start of the rupture of the second asperity. The time delay between P1 and P2 would suggest a rupture velocity of the first asperity of about 2 km/s (half the rupture length during about half the rupture duration).

The local coherency remains relatively high in the *P* coda, with specific Δ*δt* > 0 ms, probably indicating the presence of a strong scatterer NW of the source region. Stereometry results also show high coherency about 1.5 s before P1 (labeled p1 and yellow star in Figure 2 and details in the magnified trace inset) with a time shift similar to P1. This is due to a small *M*1.8 foreshock event (ID 37369693 from the QTM catalog of Ross et al. (2019), 10 June 2016 08:04:36.75; latitude = 33.43961°, longitude = −116.43604°, depth = 12.31 km) located in the hypocentral area of the mainshock. A careful inspection of the traces shows that the foreshock *P* wave is followed by another phase (labeled p2 in Figure 2) starting at 08:04:42.2 at PFO, with an opposite polarity which is interrupted by the mainshock at 08:04:42.5. Whether or not this p2 phase is generated by the *M*1.8 event, this earthquake sequence highlights the specific preparation phase of the *M*_*w*_ 5.2 Borrego Springs earthquake. This example illustrates the potential of stereometry analysis for detecting the preparatory activity of a fault at a single asperity scale, before the occurrence of intermediate‐size earthquakes. Stereometry can, therefore, contribute to the characterization of the nucleation style of such events.

### 3.2 The Le Teil Earthquake

**Figure 3:**
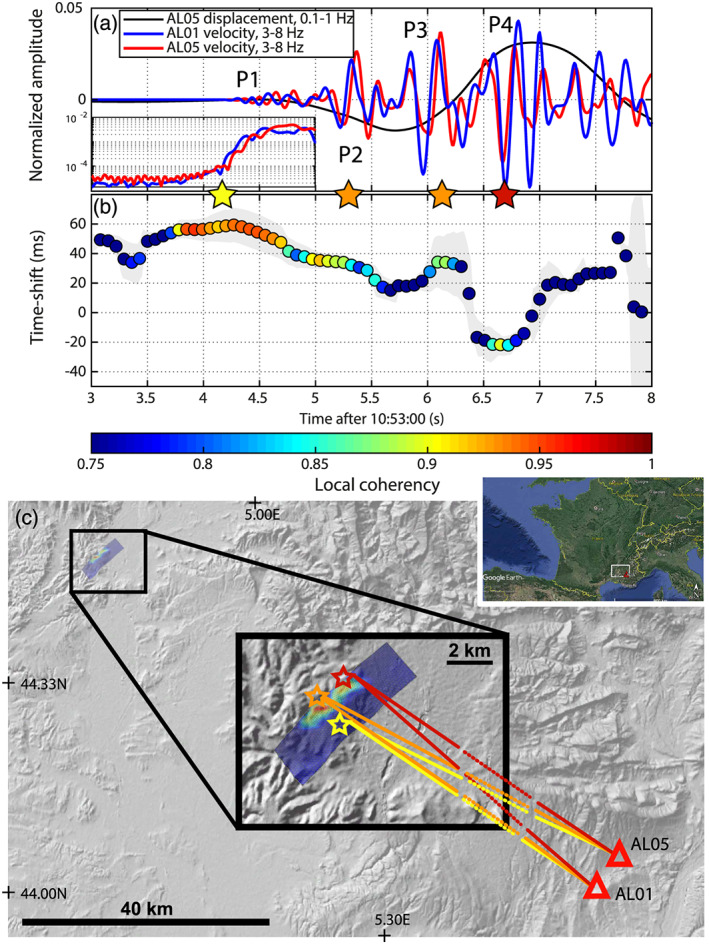
Stereometry applications: the 2019 *M*_*l*_ 5.2 Le Teil earthquake. (a) Vertical velocity traces at stations AL01 (blue) and AL05 (red) filtered between 3–8 Hz without time shift correction. The low‐frequency displacement at AL05 is shown in black. The inset shows a zoom on the envelopes of the traces in log scale at the beginning of the event highlighting the emergent nature of the waveforms. The stars show the different subevents of the seismic sequence. (b) Stereometry analysis. The gray area delineates the time shift confidence interval. (c) Map of the location of the Le Teil earthquake and the two stations used for the stereometry analysis. The gray background is a topographic image of the region. The blue and red image on the upper left corner and in the zoomed area shows the slip model obtained from InSAR data inversion (Delouis et al., 2019). The northwest side of the model is displayed along the surface rupture of the fault. The yellow, orange, and red stars and lines are interpretation of the stereometry results.

On 11 November 2019, at 10:52:45 UTC, a *M*_*l*_ 5.2 earthquake (SISMOAZUR ID: oca2019wcnm) broke the Rouvière fault in southeast France, close to the Rhone valley. The mechanism was mostly in thrust with a 45° strike, 58° dip, and 82° rake, reactivating an ancient normal fault. As of the time of the writing of this report, the position of the epicenter (∼Lat = 44.62°, ∼Lon = 4.67°) is only known with spatial uncertainty smaller than 1–2 km (Delouis et al., 2019), the depth, however, is fairly well constrained to be about 2 km depth or shallower, within a thick layer of Cretaceous marl, overlaid by an eroded limestone layer. This shallow source resulted in significant surface deformations including a surface expression of the fault rupture. This permits to use of InSAR data to invert for the slip distribution on the fault plane (Delouis et al., 2019). The resulting slip model is shown in Figure 3c). The slip is distributed onto two main patches with amplitude reaching up to 30 cm at the center of the dark‐red patch and 20 cm on the northern patch. The total rupture length was about 4 km and most of the slip was confined between 1 km depth and the surface.

Available permanent seismic stations in the vicinity of the earthquake did not provide any usable pair of stations for a stereometry analysis. Only two stations (AL01, lat = 44.00545°, lon = 5.74582°; AL05, lat = 44.05611°, lon = 5.78751°) from a temporary seismic network, deployed about 90 km southeast from the epicentral area, were found to be suitable for the measurements. The distance between the stations is 6.54 km. The differential epicentral distance at the two stations, Δ*D*, is about 100 m, with a 102 km epicentral distance. This configuration produces a very high coherence between the event waveforms for a wide frequency range. From the InSAR data, we can estimate the fault length to be about 4 km, therefore, we need wavelengths smaller than 2 km to be able to resolve detailed features along the fault plane. At this epicentral distance, the average *Vp* along the ray is about 5 km/s, so we need to analyze the seismograms at frequencies higher than 3 Hz. We find that the best SNR is achieved between 3 and 8 Hz (Figure 3a). In this frequency band, the seismograms have a coherency larger than 0.75 for the first 3 s of the *P* wave. The *P* onset is emergent, starting around 10:53:04.2 at the two stations (inset in Figure 3a). This first high coherency, low‐amplitude wave train (P1, yellow star in Figure 3a) is followed by three larger pulses (P2 and P3, orange stars and P4, red star) for which the coherency is slightly smaller, around 0.85. The time shift between AL01 and AL05 decreases along time and delineates three different plateaus: P1 with a coherency larger than 0.92 and d*t*_*P* 1_ = 0.06 s, P2 and P3 with similar coherency (0.85–0.9) and d*t*_*P* 2_ = d*t*_*P* 3_ = 0.035 and P4 with a 0.85 coherency and d*t*_*P* 4_ = −0.02. These observations suggest that the early sources of energy (P1 to P3) are relatively closer to station AL01 than AL05 but that the source of P4 is closer to AL05 than AL01.

InSAR data characterized two main patches of slip close to the surface. Contrarily to the Borrego Springs earthquake, the identification and association of each *P* wave pulse to a specific asperity on the fault is less evident. The latest analysis locates the hypocenter below the main slip patch, at 1–2 km depth (Delouis et al., 2019). The rupture would then migrate toward the surface. The interpretation of the stereometry results indicates that P1, generated at the hypocenter, has a high coherency because the waves propagated from a deeper depth and did not encounter heterogeneities in the near surface on the source side. The time shift d*t*_*P* 1_ is the result of the geometry of the station's position with respect to the location of the hypocenter. The coherency of P2 and P3, is slightly lower because they are most likely generated by the large slip patch at the surface. The waves are crossing more heterogeneous media resulting in less coherent arrivals. The smooth transition of the time shifts from P1 to P2 could be due to the evolution of the ray parameter (Equation 1) from the hypocenter to the rupture closer to the surface. The time shifts of P1, P2, and P3 are positive, meaning that the location of the source is closer to AL01 than AL05. The exact nature of P2 and P3 is not understood for the moment. P4 has a slightly lower coherency, which could be due to both its near‐surface source and the possible contamination by scattered waves from the previous sources. The negative time shift of P4 indicates that its source is now closer to AL05 than AL01. This is compatible with the location of the northern slip patch. The stereometry results seem to be compatible with a scenario where the hypocenter is at depth below the main slip patch, then the rupture propagates to the main slip patch, and finally to the secondary northern slip patch. From the stereometry measurements, using a ray parameter *p* = 0.1818s/km, we estimate the horizontal rupture length and velocity using the time shift difference *dt*_*P* 4_ − *dt*_*P* 2_ = −54 ± 7ms (neglecting the rupture from the hypocenter to the surface) and the pulses traveltime difference *t*_*P* 4_ − *t*_*P* 2_ = 1.48s. We find a rupture length of 4.75 ± 0.6 km and a rupture velocity of 2.8 ± 0.3 km/s. The rupture length estimated from stereometry matches very closely the total horizontal length of the slip distribution seen with InSAR (Figure 3c). The estimated rupture velocity, about 77% of the local *Vs* (C. Cornou, personal communication, June 8 2020) strongly depends on the interpretation of the nature of each high‐frequency *P* wave pulse. If the arrival‐time *t*_*P* 3_ of the third pulse is used in the calculation, the rupture length is unchanged (similar time shift) but the rupture velocity increases dramatically to about 5 km/s, which is highly unlikely. The complete interpretation of the stereometry analysis will only be possible if we can identify the nature of each high‐frequency pulse, which will require a more in‐depth analysis of this event, beyond the scope of this paper.

## 4 Discussions and Conclusion

Seismic stereometry can be seen as a generalization of earthquake location by double‐difference methods (Waldhauser & Ellsworth, 2000) applied to the different high‐frequency pulses generated during an earthquake source. Instead of correlating two events at one station, stereometry uses the correlation of the same event at two neighboring stations to retrieve the timing and relative locations of the source of each pulse, with respect to the first one. As a double‐difference method, seismic stereometry can resolve very fine spatiotemporal details of earthquake ruptures. Because it can inform about the direction of propagation of the rupture, stereometry can be used to solve the fault‐plane ambiguity from centroid moment tensor (Chen et al., 2010). It can also help to detect badly located earthquakes in catalogs, if pairs of seismograms at neighboring stations do not overlap as expected from the cataloged location of the events.

The main practical limitation of the method is, for the moment, the quite rare occurrence of neighboring pairs of stations in the right configuration for the event. In California, for instance, we found only 50 events (*M*_*w*_ 4.5–6.5) between 2006 and 2020 for which at least one pair of stations was suitable for stereometry. The current densification of permanent seismic networks and the emergence of denser and denser arrays will permit us to analyze more and more earthquakes using stereometry. The interpretation of the stereometry results can also be limited if one misinterprets the origin and nature of the analyzed high‐frequency *P* wave pulses. The interpretation of the timing, nature, and localization of the different *P* wave pulses can be refined using 3‐D velocity models and source kinematic models (Yin & Denolle, 2019). In general, additional external information might be needed to solve some interpretation ambiguities.

Nevertheless, if one earthquake can be studied with stereometry on a pair of stations and if long time series are available, it becomes possible to use stereometry on continuous records to look for time shift and coherency patterns that match the ones of the main event. By doing so, similar to the approach of Hawthorne and Ampuero (2018); Tape et al. (2018), we can trace back the activity history of the portion of the fault that generated the main event and potentially uncover precursory behaviors on this segment. This approach is complementary but different from template‐matching analysis (Ross et al., 2019) because we are looking for high coherency between two stations and not between two events. This way, signals very different from the main event can be detected and traced back to its location, if the time shift between the two stations is the same. With that in mind, it would be interesting to scan seismic catalogs to see if pairs of seismic stations are available for stereometry analysis and look for potential precursory patterns before small to intermediate earthquakes. Focusing on induced seismicity (Ellsworth, 2013) could reveal details about the triggering mechanisms of such earthquakes and the relationship to fluid injection in the crust. At a larger scale, stereometry can also be used to study the source complexity of large earthquakes, provided that the station geometry and the earthquake are in the right configuration. Records from past earthquakes (Ruiz et al., 2011) could be revisited to infer finer source mechanisms when other geophysical data are missing. On a smaller scale, using stereometry on borehole data should give access to source kinematics of even smaller earthquakes which could help resolve the question of earthquake source self‐similarity across all magnitudes. In general, stereometry can be used at any scale, with several pairs of stations in an array and can potentially provide a higher time‐frequency resolution than standard back‐projection methods.

## Conflict of Interest

The authors declare that they have no competing interests.

## Data Availablity Statement

The PY network data are from UC San Diego (2014): Piñon Flats Observatory Array. International Federation of Digital Seismograph Networks. Dataset/Seismic Network. https://doi.org/10.7914/SN/PY. The PFO data are from UC San Diego (1982): ANZA Regional Network. International Federation of Digital Seismograph Networks. Dataset/Seismic Network. https://doi.org/10.7914/SN/AZ. The data from stations AL01 and AL05 will be available via the RESIF portal under FDSN code XW after 2022. We used the quaternary fault database for the United States from the USGS (https://earthquake.usgs.gov/hazards/qfaults/). The cross‐wavelet transform function developed by Mao et al. (2019) and used in this study is available at https://github.com/shujuanmao/dt‐wavelet. Seismic data from AZ and PY networks were retrieved using obspyDMC (Hosseini & Sigloch, 2017). Figures 2 and 3 were made with GeoMapApp (www.geomapapp.org) / CC BY.

## Supporting information



Supporting Information S1
