# Supplementary material for: Seismic Stereometry Reveals Preparatory Behavior and Source Kinematics of Intermediate‐Size Earthquakes
Source: Geophys Res Lett. 2020 Sep 2;47(17):e2020GL088563. doi: 10.1029/2020GL088563 (PMC7583370; doi:10.1029/2020GL088563)
Supplement: Supplementary file 1 — Supporting Information S1 [file GRL-47-e2020GL088563-s001.pdf]

Supporting Information for "Seismic Stereometry reveals preparatory behavior and source kinematics of intermediate-size earthquakes"

A. Mordret[1], F. Brenguier[1], M. Causse[1], P. Boué[1], C. Voisin[1], I. Dumont[1,2],

F. L. Vernon[3] and J.P. Ampuero[4]

[1]Univ. Grenoble Alpes, Univ. Savoie Mont Blanc, CNRS, IRD, IFSTTAR, ISTerre, 38000 Grenoble, France

[2]TOTAL CSTJF, avenue Larribau, 64018 Pau, France

[3]Scripps Institution of Oceanography, University of California, San Diego, 9500 Gilman Drive #0225, La Jolla, California 92093, U.S.A.

[4]Université Côte dAzur, IRD, Observatoire de la Côte d'Azur, CNRS, Géoazur, Valbonne Sophia Antipolis, France

Contents of this file

1. Text S1 to S3
2. Figures S1 to S24
3. Tables S1 to S2

Introduction

Corresponding author: Aurelien Mordret, aurelien.mordret@univ-grenoble-alpes.fr

1. Text S1: Derivation of the rupture length and rupture velocity from differential travel-time measurements

Let the coordinates of the hypocenter be X_h, Y_h and Z_h (positive to the east, north, and down, respectively) and $\mathbf{r} = (\delta x_r, \delta y_r, \delta z_r)$ the vector linking the hypocenter to the end of the rupture. The strike of the fault is θ_f and $\delta\tau \neq 0$ is the rupture time. Similarly, let the vector linking the pair of stations be $\mathbf{S} = (\delta x_s, \delta y_s, \delta z_s)$ and the azimuth of the station pair θ_s. The azimuth from the center of the rupture to the station pair is defined as $A_{rs} = \theta_f + \psi$. If we assume that the epicentral distance D is much larger than the rupture length and much larger than the two stations separation, we can use a double-difference approach (Got et al., 1994; Waldhauser & Ellsworth, 2000) and define the travel-time difference between the first and last pulses of the rupture recorded at station s_1 as:

$$\Delta t_r^{s_1} = -\frac{1}{V_h}(\mathbf{r} \cdot \mathbf{n}_{rs_1}) + \delta\tau \,, \tag{1}$$

where $\mathbf{n}_{rs_1} = (\sin A_{rs_1} \sin\gamma_{rs_1},\, \cos A_{rs_1} \sin\gamma_{rs_1},\, \cos\gamma_{rs_1})$ is the unit vector of the ray from the barycenter of the rupture to the station s_1, with A_{rs_1} the azimuth to the station and γ_{rs_1} the incidence angle at the hypocenter. The velocity in the hypocentral region is V_h is assumed constant and $(\cdot)$ defines the dot-product between two vectors. The same relation holds for the second station s_2. We can therefore estimate the rupture time as:

$$\delta\tau = \frac{\Delta t_r^{s_1} + \Delta t_r^{s_2}}{2} + \frac{\mathbf{r} \cdot \mathbf{n}_{rs}}{V_h} \,, \tag{2}$$

with $\mathbf{n}_{rs} = (\sin\psi \sin\gamma_{rs},\, \cos\psi \sin\gamma_{rs},\, \cos\gamma_{rs})$, assuming $\gamma_{rs_1} \simeq \gamma_{rs_2} \simeq \gamma_{rs}$.

Using the seismic reciprocity principle, we can write the travel-time difference of the first pulse of the rupture between station s_1 and s_2 as:

$$\Delta t_{s_1 s_2}^{firstpulse} = -\frac{1}{V_{sta}} (\mathbf{S} \cdot \mathbf{n}_{sh}) \, , \tag{3}$$

where $\mathbf{n}_{sh} = (\sin A_{sh} \sin \gamma_{sh}, \, \cos A_{sh} \sin \gamma_{sh}, \, \cos \gamma_{sh})$ is the unit vector of the ray from the barycenter of the station pair to the hypocenter, with A_{sh} the azimuth to the hypocenter and γ_{sh} the incidence angle at the stations. The velocity below the stations V_{sta} is assumed constant. Similarly, the travel-time difference between station s_1 and s_2 of the pulse generated at the end of the rupture is:

$$\Delta t_{s_1 s_2}^{lastpulse} = -\frac{1}{V_{sta}} (\mathbf{S} \cdot \mathbf{n}_{se}) \, , \tag{4}$$

where $\mathbf{n}_{se} = (\sin A_{se} \sin \gamma_{se}, \, \cos A_{se} \sin \gamma_{se}, \, \cos \gamma_{se})$ is the unit vector of the ray from the barycenter of the station pair to the end of the rupture, with A_{se} the azimuth to the end of the rupture and γ_{se} the incidence angle at the stations. Lets $\alpha \neq 0$, positive clockwise, be the angle under which the pair of stations sees the length of the rupture L_r (Fig. 1d), such that $A_{se} = A_{sh} + \alpha$. In this case, we have $L_r \simeq D\alpha/sin(\psi)$ when $L_r \ll D$. To estimate L_r, let us subtract eq. 3 from eq. 4 to get $\Delta t_{s_1 s_2}^{lastpulse} - \Delta t_{s_1 s_2}^{firstpulse} = \Delta \delta t$ the difference of time-shift between the two stations between the last pulse and the first pulse of the rupture. Again, assuming that α is small and that the two stations are close enough so that $\gamma_{sh} \simeq \gamma_{se}$ and $A_{sh} \simeq A_{rs} + 180^o$, we find after some algebra:

$$L_r \simeq D|\alpha| = \frac{D|\Delta\delta t|}{p\sin(\psi)(\delta x_s \cos A_{rs} - \delta y_s \sin A_{rs})}\,, \tag{5}$$

where p is the ray parameter between the rupture and the stations. The sign of $\Delta\delta t$ and α gives the direction of propagation: $\Delta\delta t > 0$ means that the rupture is getting closer to station i or away from station j. If the rupture propagates mostly horizontally, we have $|\mathbf{r}| \simeq L_r$ and the rupture velocity V_r is obtained from eq. 2:

$$V_r = \frac{L_r}{\delta\tau} \simeq \frac{2L_rV_h}{V_h(\Delta t_r^i + \Delta t_r^j) + 2L_r\cos(\psi)}\,. \tag{6}$$

2. Text S2: Effects of the radiation pattern on the stereometry results

Stereometry works only if the coherency between the two stations is high. So, if because of the radiation pattern, the two stations are on both sides of a nodal plane, it is likely that the coherency between the waveforms is low and that station pair is rejected from further analysis. However, if it is recognized that the two waveforms are emerging from two different sides of a nodal plane, i.e., one is the negative version of the other, then stereometry can be used on the flipped versions of the waveforms. Nevertheless, given that we use stations separated by distances on the order of a wavelength and in a finite frequency framework, we do not expect to encounter this issue very often.

3. Text S3: Stereometry measurements on the Pinon Flat array

This supporting information shows the application of seismic stereometry on all 17 suitable pairs of stations from the Pinon Flat array. The Figures S8 to S24 show the stereometry measurements for each of the 17 pairs. Each figure presents 3 panels: the top

left one shows the ground velocity waveforms from station 1 (in blue) and station 2 (in red) filtered in the 5-12 Hz frequency band using an order 2 minimum phase Butterworth filter. In black is shown the displacement at station 1 filtered in the 0.1-3 Hz frequency band. The top right panel shows a map of the two stations, with their names color-coded in blue for station 1 and in red for station 2. The cyan arrow points toward the epicenter of the Borrego Spring earthquake. The magenta arrow points toward the end of the rupture of the Borrego Springs earthquake estimated by Ross et al. (2017). The green arrows points toward the range of positions for the end of the rupture as estimated with stereometry, taking the uncertainties into account. When the green arrows point to the side of station 1, we expect the time-shift between the two traces to be positive. When they point toward the side of station 2, we expect the time-shift to be negative. The closer the green arrows are to the bisector of the two stations (perpendicular black line), the smaller the time-shift is expected to be. The bottom left panel shows the stereometry measurements as described in the main text. The time-shift difference between the third pulse (mean time-shift in the window 08:04:43.45 to 08:04:43.65) and the first pulse (mean time-shift in the window 08:04:42.4 to 08:04:42.6) is shown at the bottom of the panel and is used to estimate the rupture velocity and length as described in the main text. The rupture parameters as well as other geometrical parameters used to estimate them are shown at the bottom right.

Table S1 lists the seismic stations and their coordinates used for the Borrego Springs earthquake.

References

Got, J.-L., Fréchet, J., & Klein, F. W. (1994). Deep fault plane geometry inferred from multiplet relative relocation beneath the south flank of Kilauea. *Journal of Geophysical Research: Solid Earth*, *99*(B8), 15375–15386.

Ross, Z., Kanamori, H., & Hauksson, E. (2017). Anomalously large complete stress drop during the 2016 Mw 5.2 Borrego Springs earthquake inferred by waveform modeling and near-source aftershock deficit. *Geophysical Research Letters*, *44*(12), 5994–6001.

Waldhauser, F., & Ellsworth, W. L. (2000). A double-difference earthquake location algorithm: Method and application to the northern Hayward fault, California. *Bulletin of the Seismological Society of America*, *90*(6), 1353–1368.

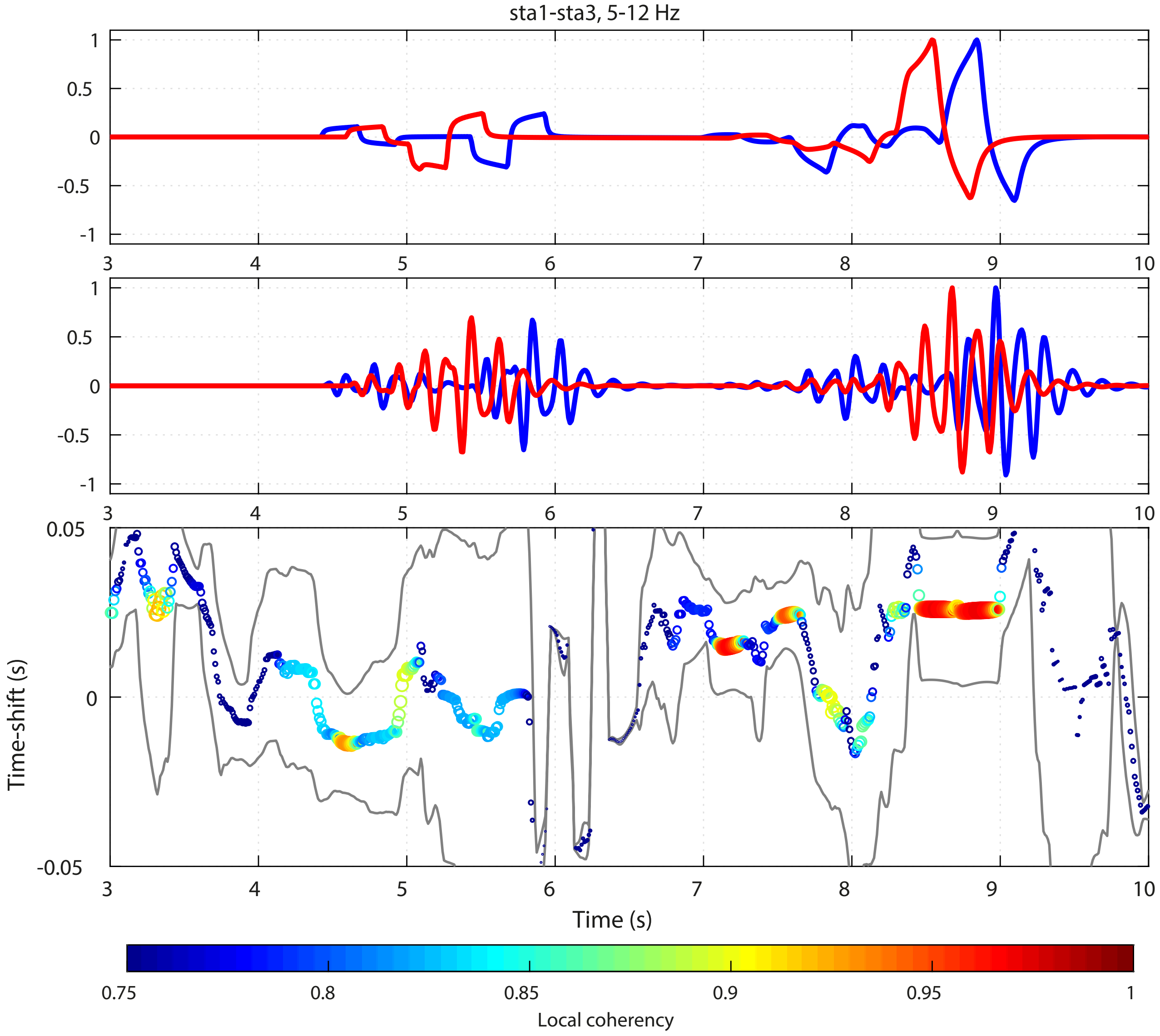

Figure S1. Synthetic case: stereometry analysis for station pair 1-3.

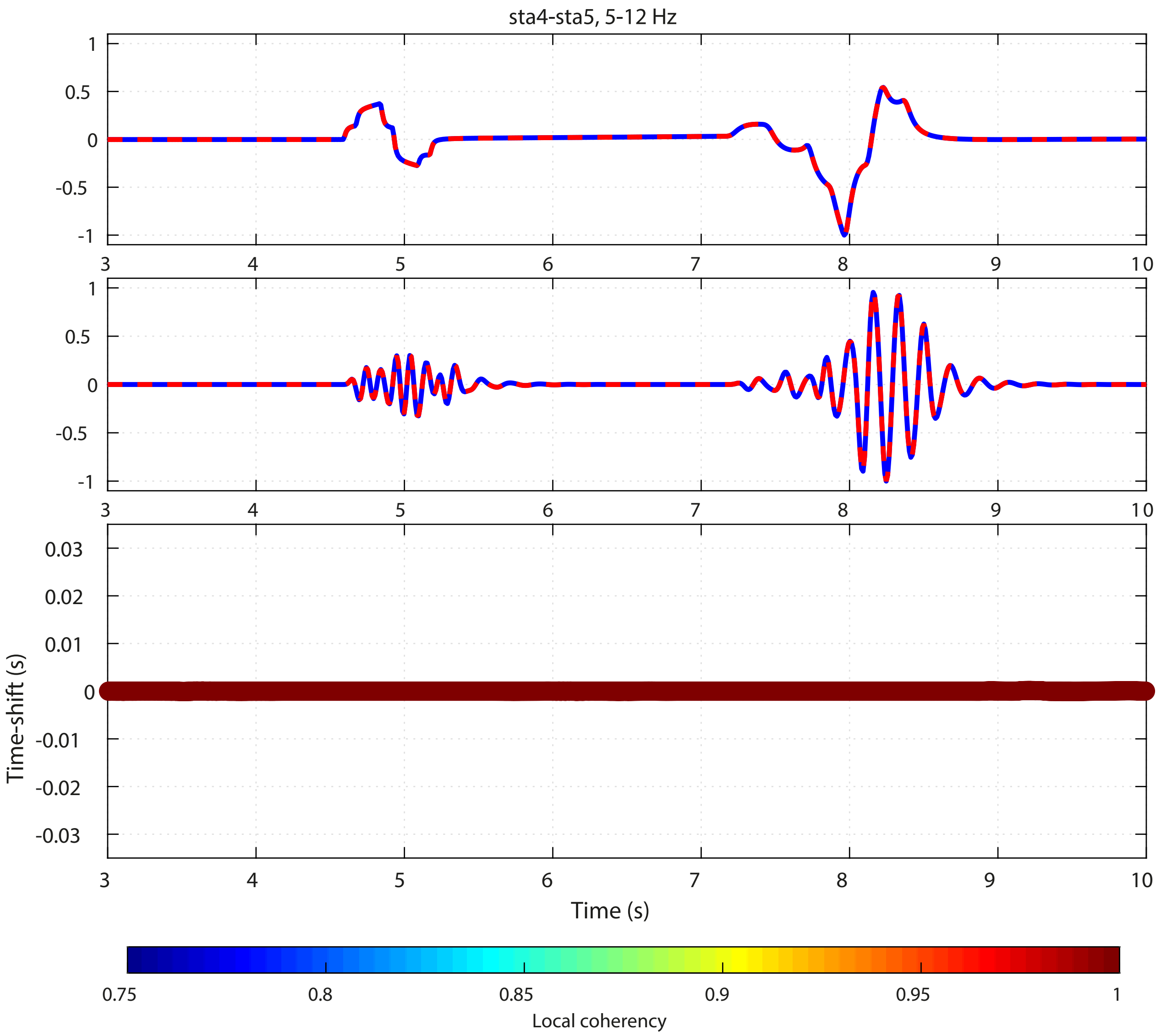

Figure S2. Synthetic case: stereometry analysis for station pair 4-5.

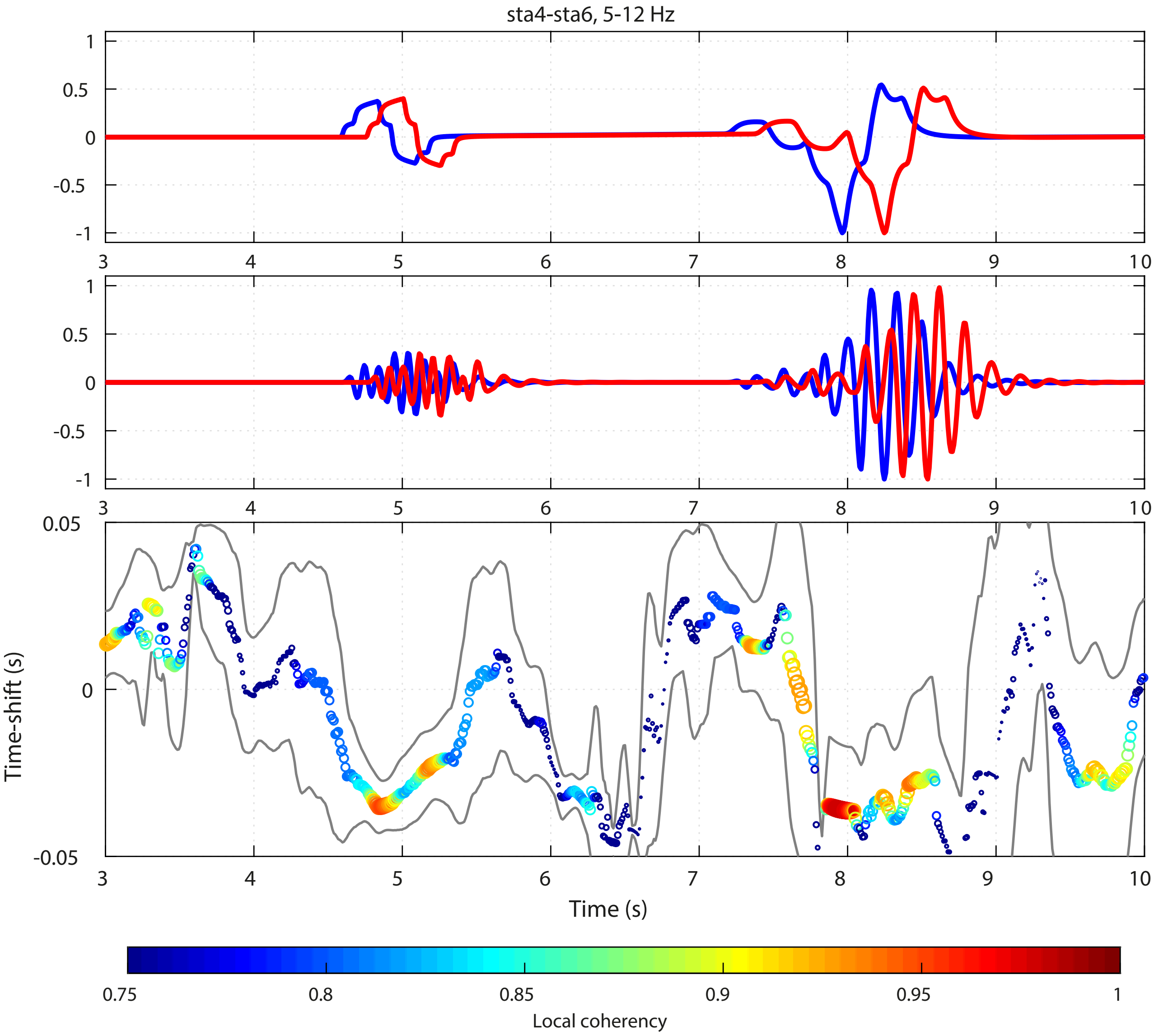

Figure S3. Synthetic case: stereometry analysis for station pair 4-6.

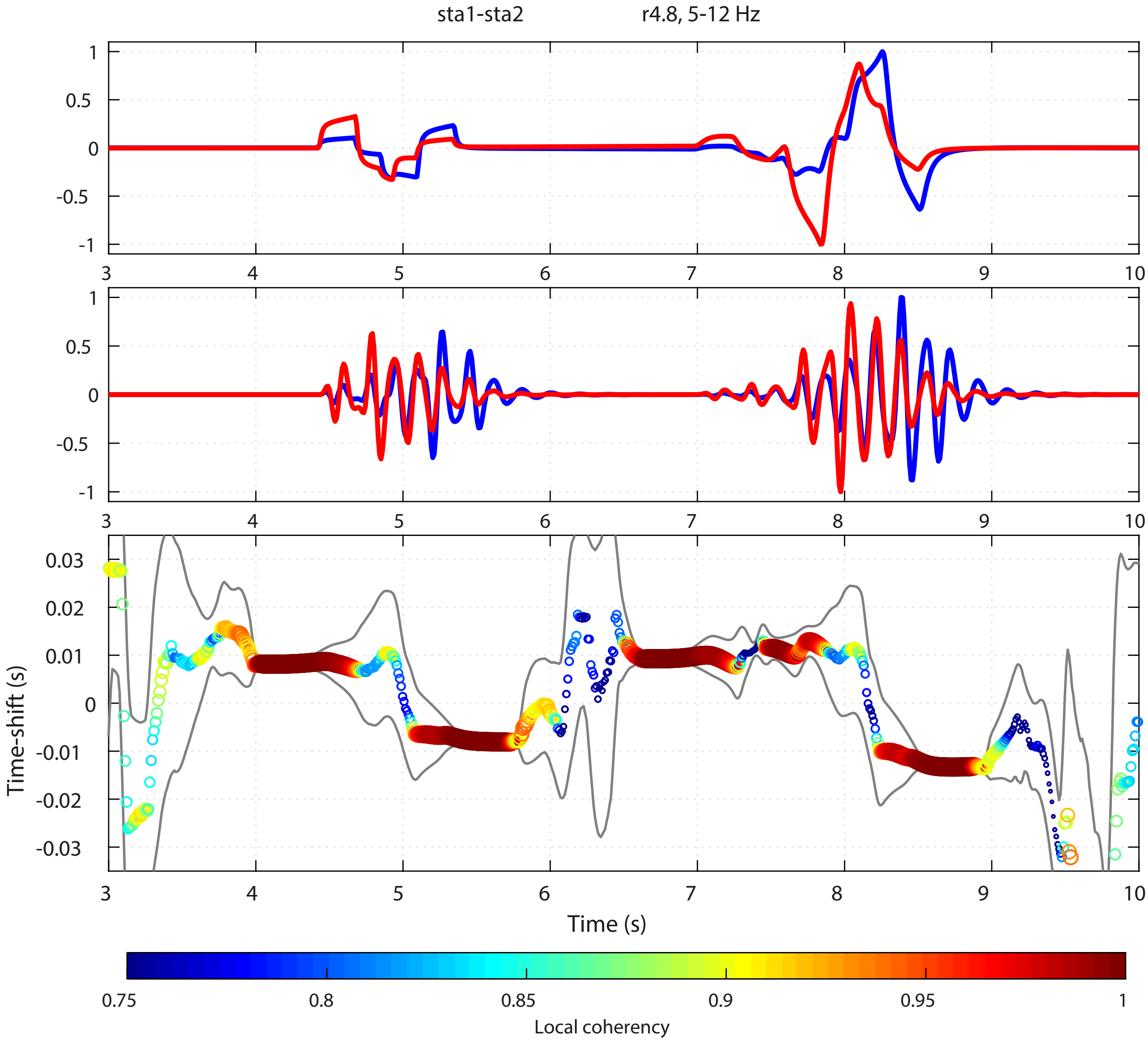

Figure S4. Synthetic case: stereometry analysis for station pair 1-2 with a rupture velocity $V_r = 4.8$ km/s.

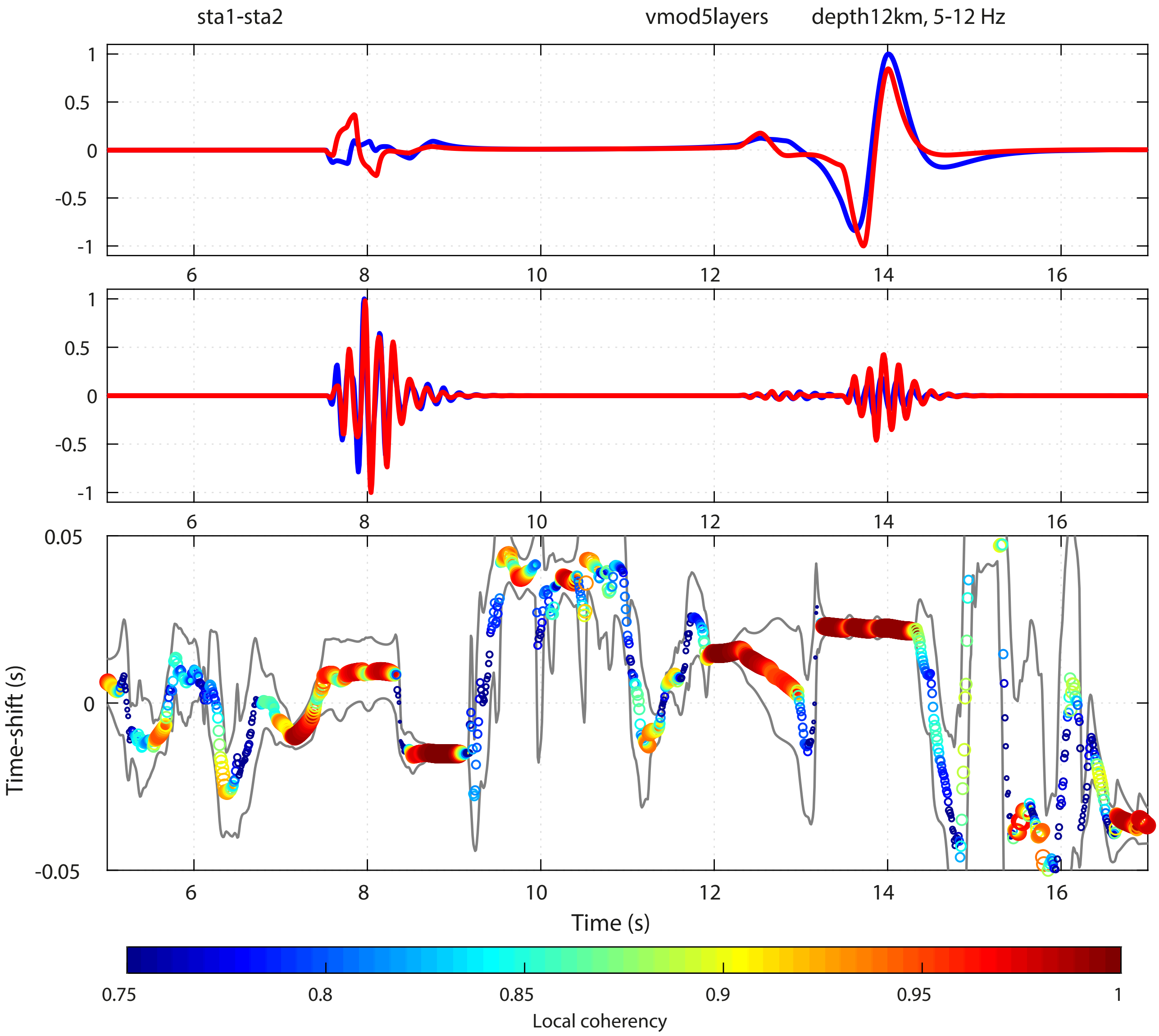

Figure S5. Synthetic case: stereometry analysis for station pair 1-2 within a 5 layer model and hypocenter depth at 12 km.

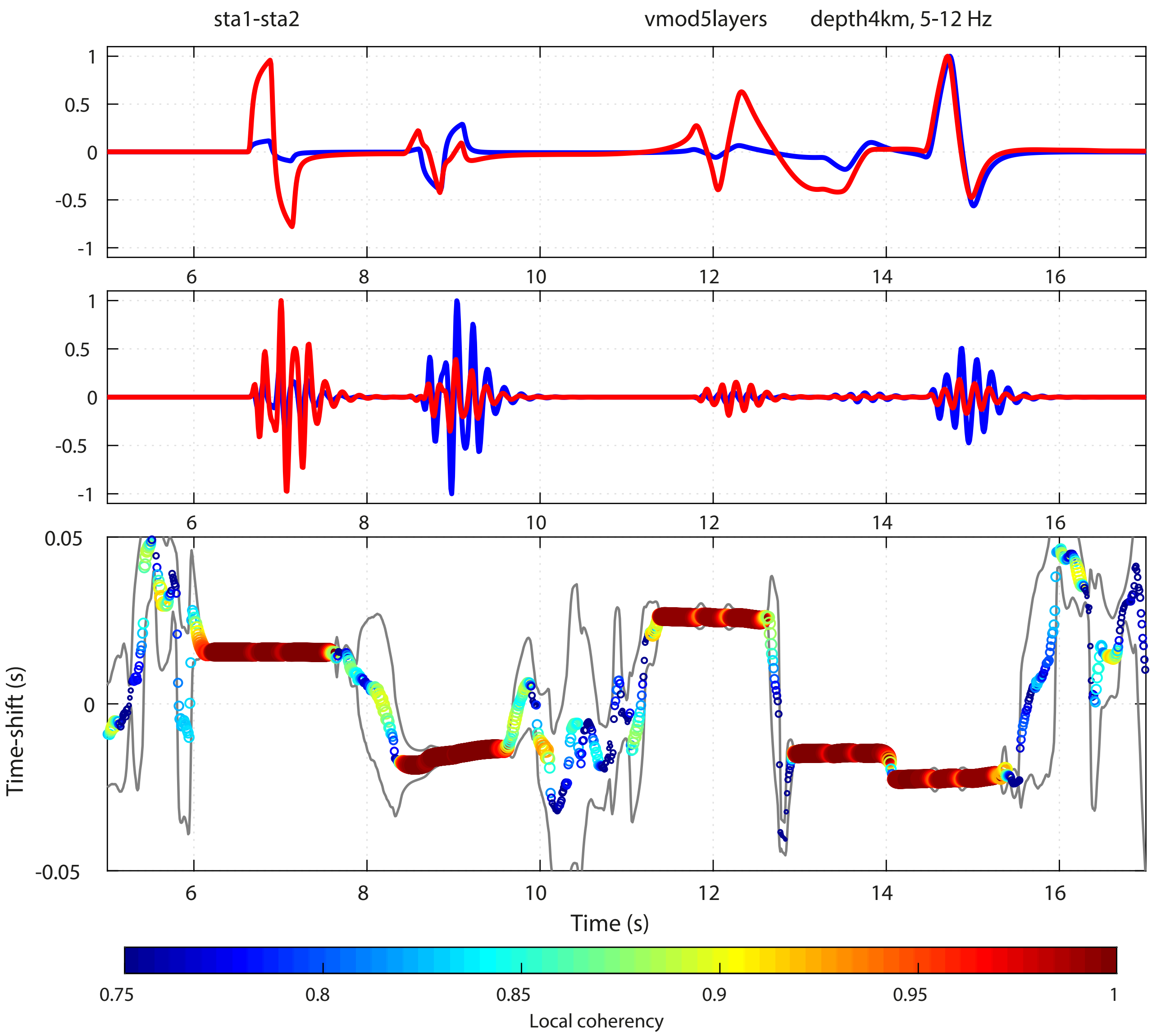

Figure S6. Synthetic case: stereometry analysis for station pair 1-2 within a 5 layer model and hypocenter depth at 4 km.

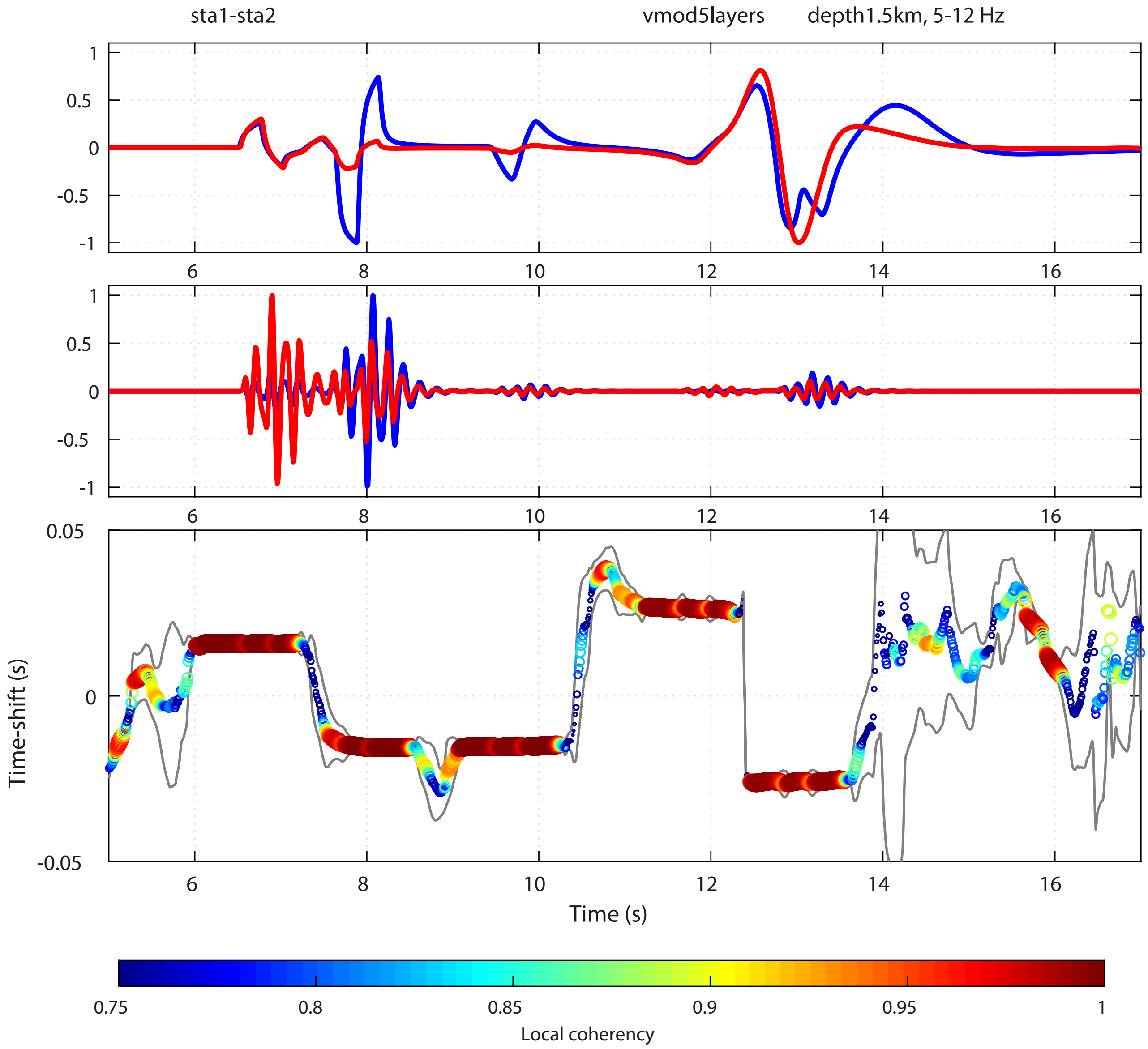

Figure S7. Synthetic case: stereometry analysis for station pair 1-2 within a 5 layer model and hypocenter depth at 1.5 km.

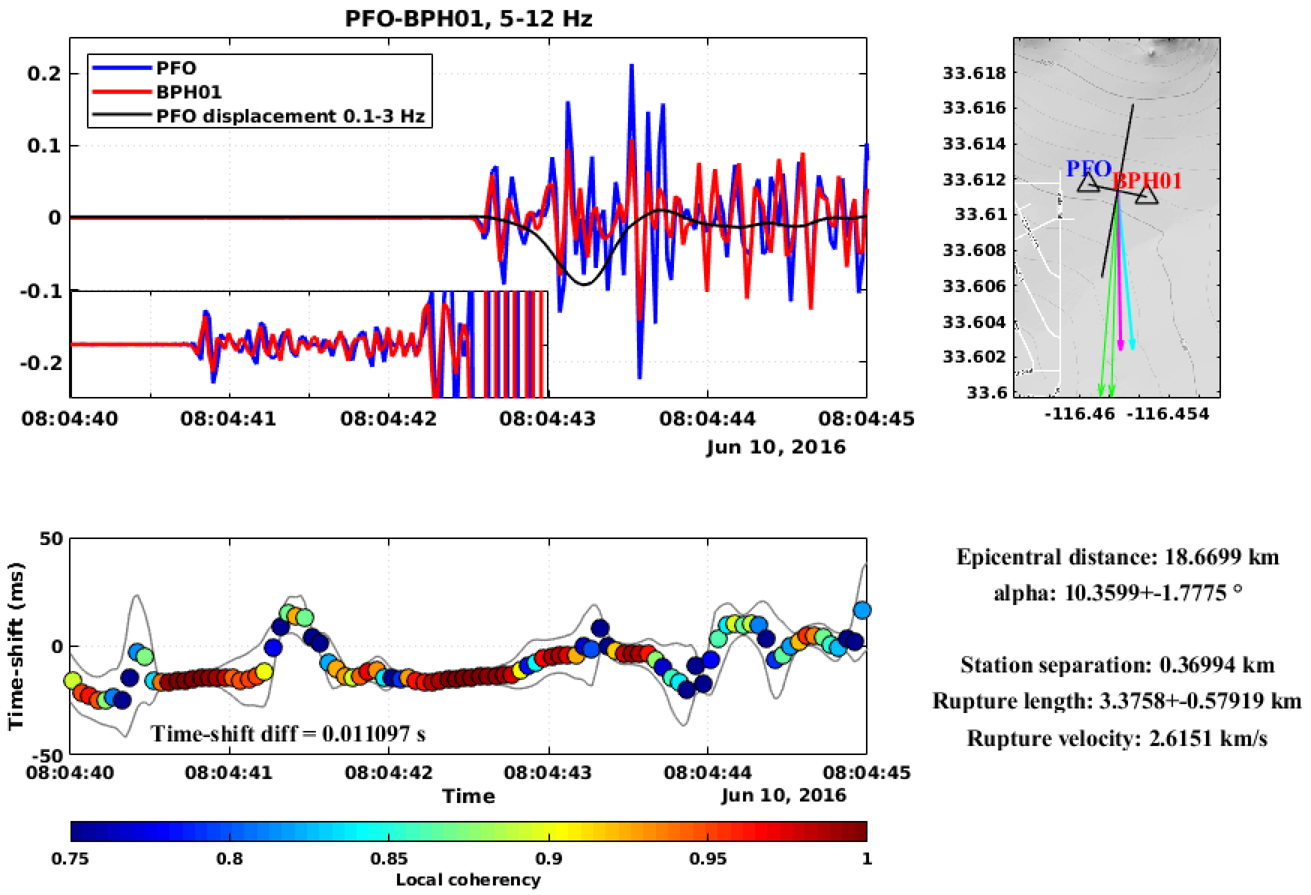

Figure S8. Stereometry analysis for station pair PFO-BPH01.

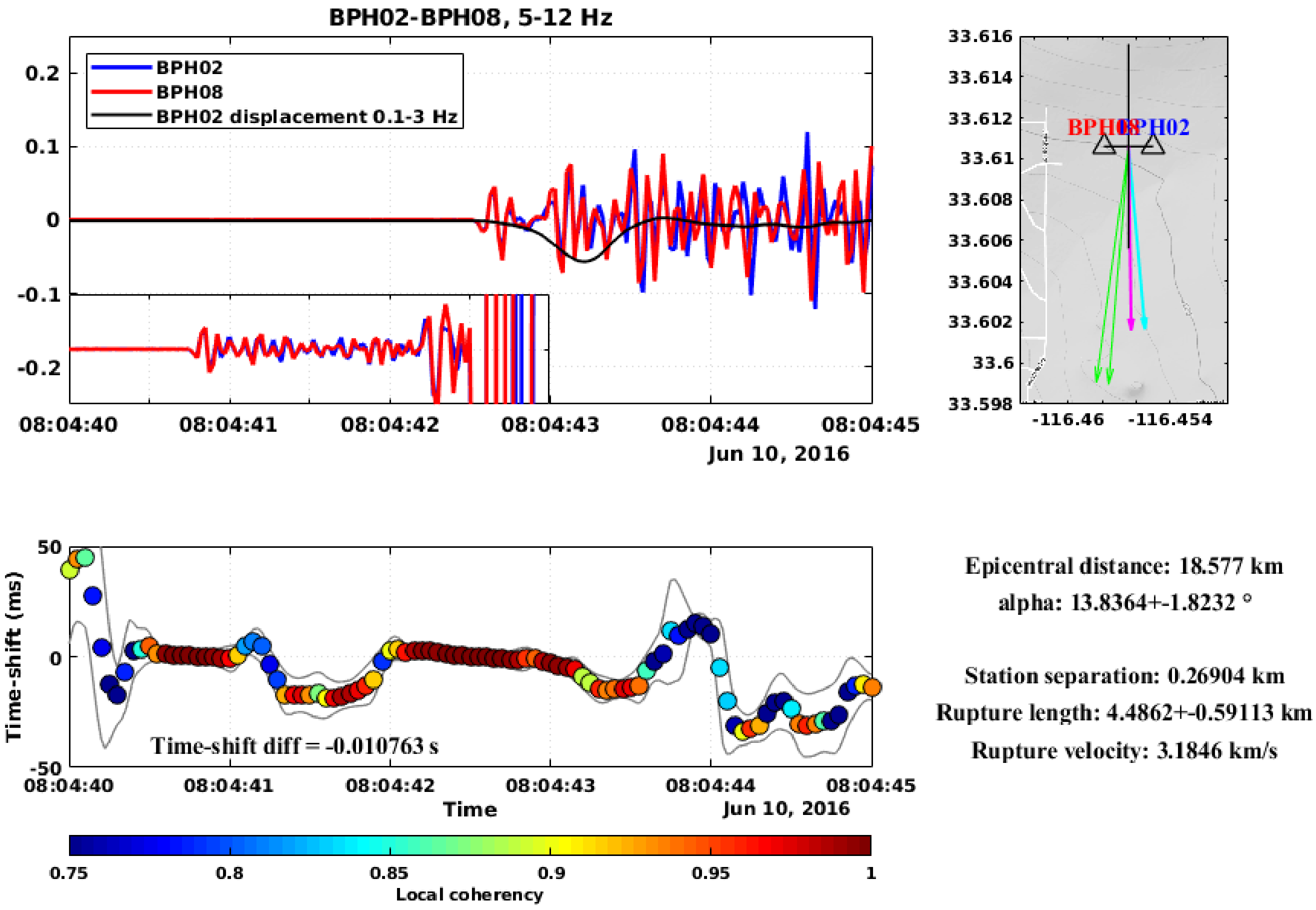

Figure S9. Stereometry analysis for station pair BPH02-BPH08.

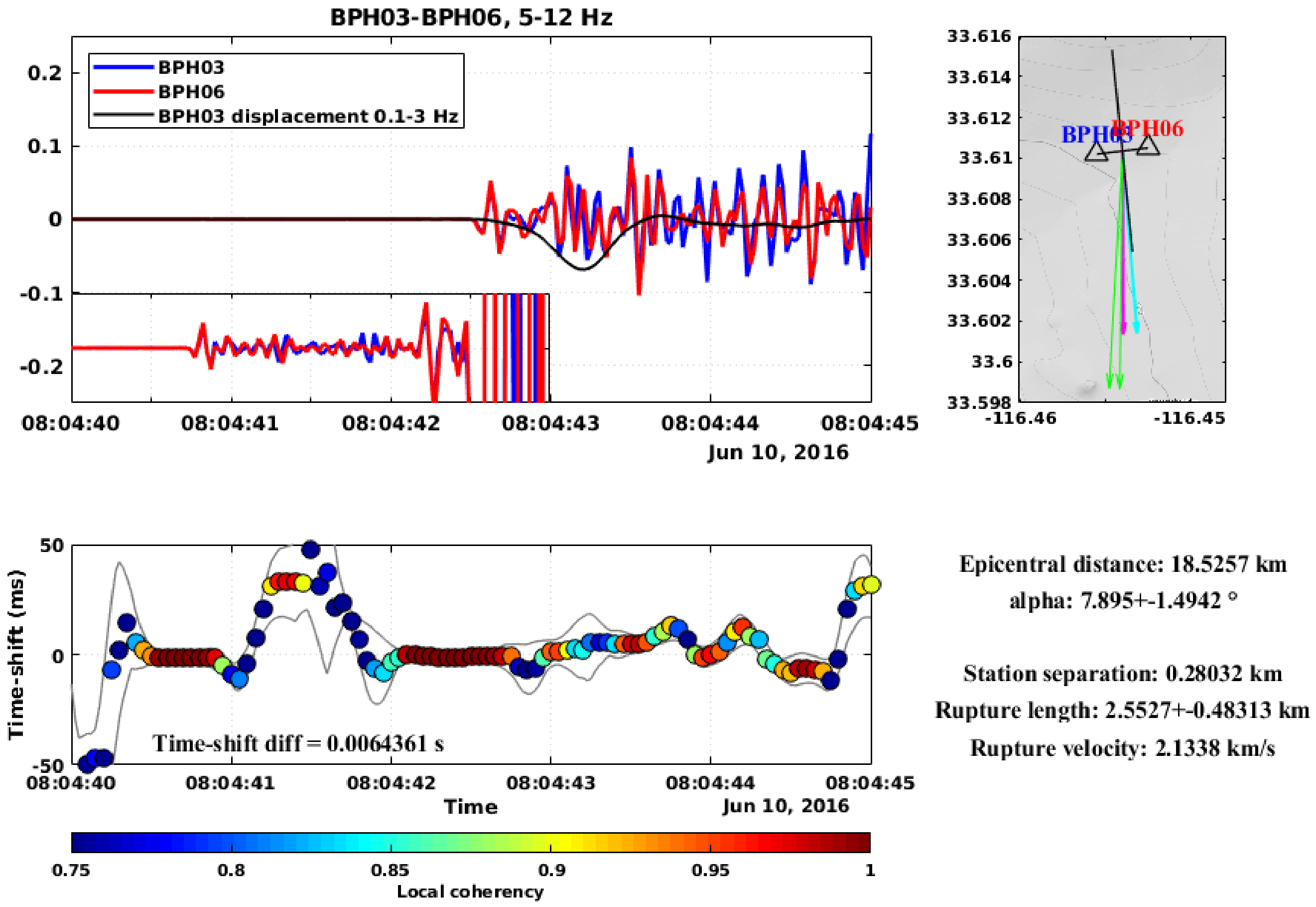

Figure S10. Stereometry analysis for station pair BPH03-BPH06.

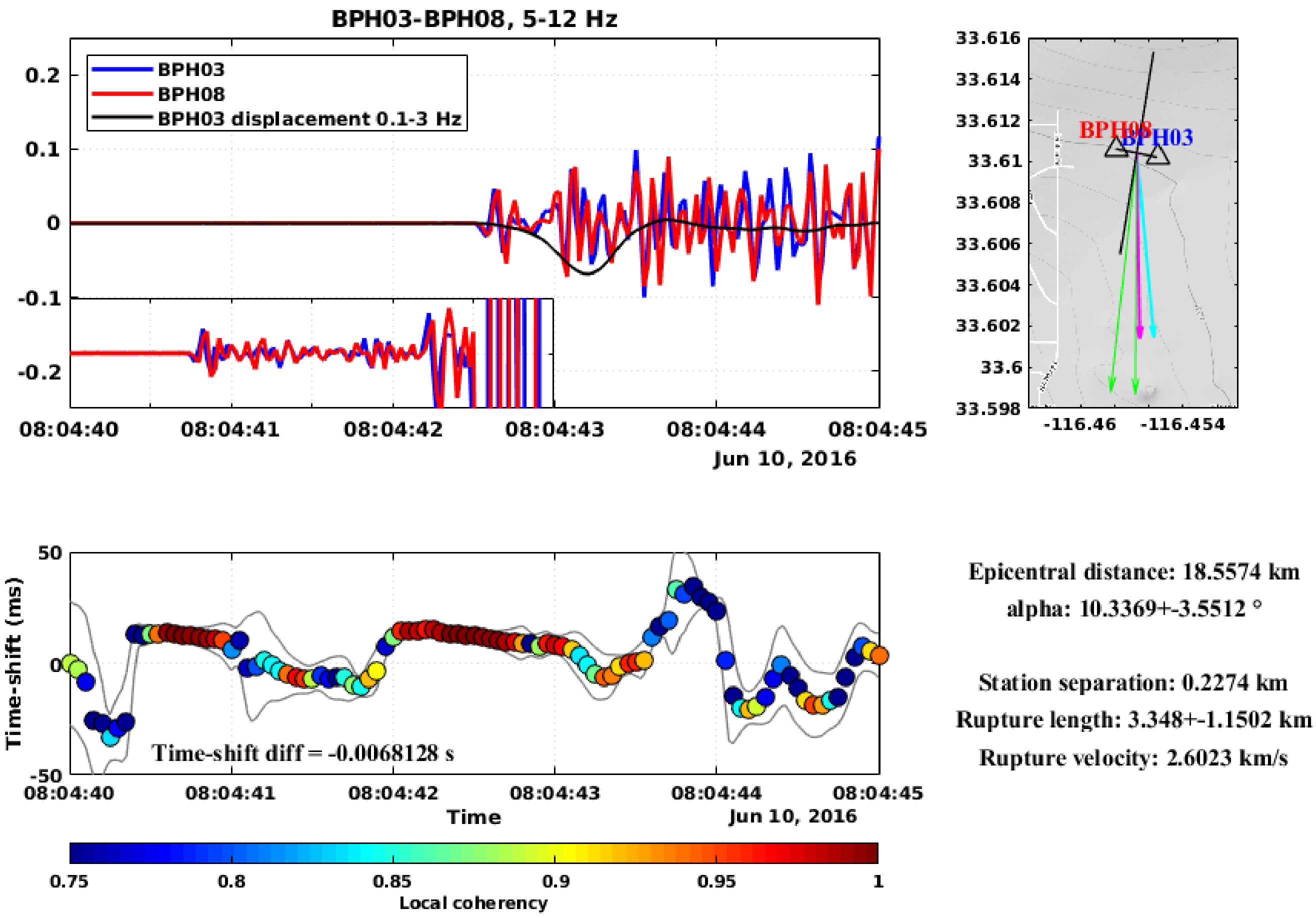

Figure S11. Stereometry analysis for station pair BPH03-BPH08.

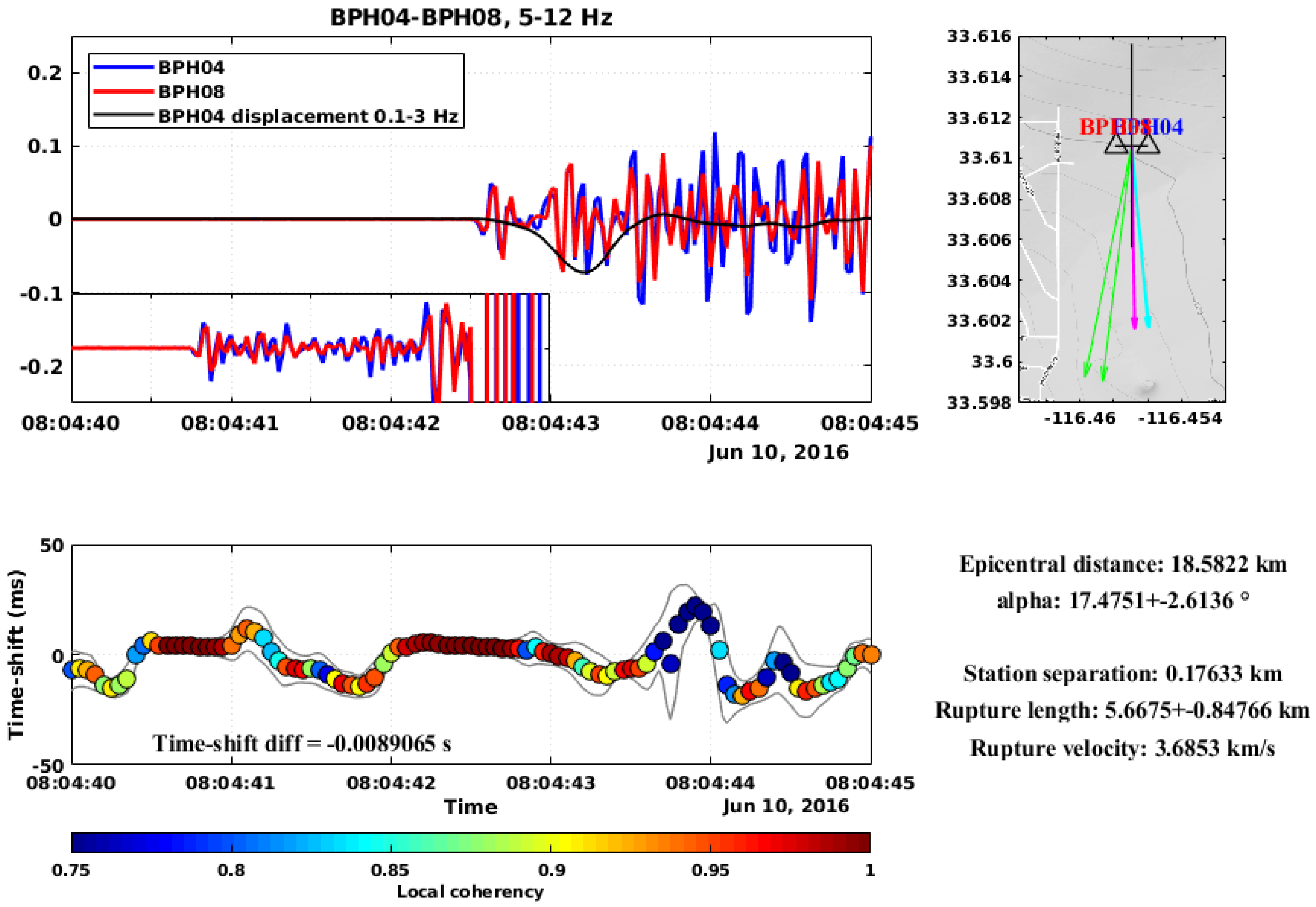

Figure S12. Stereometry analysis for station pair BPH04-BPH08.

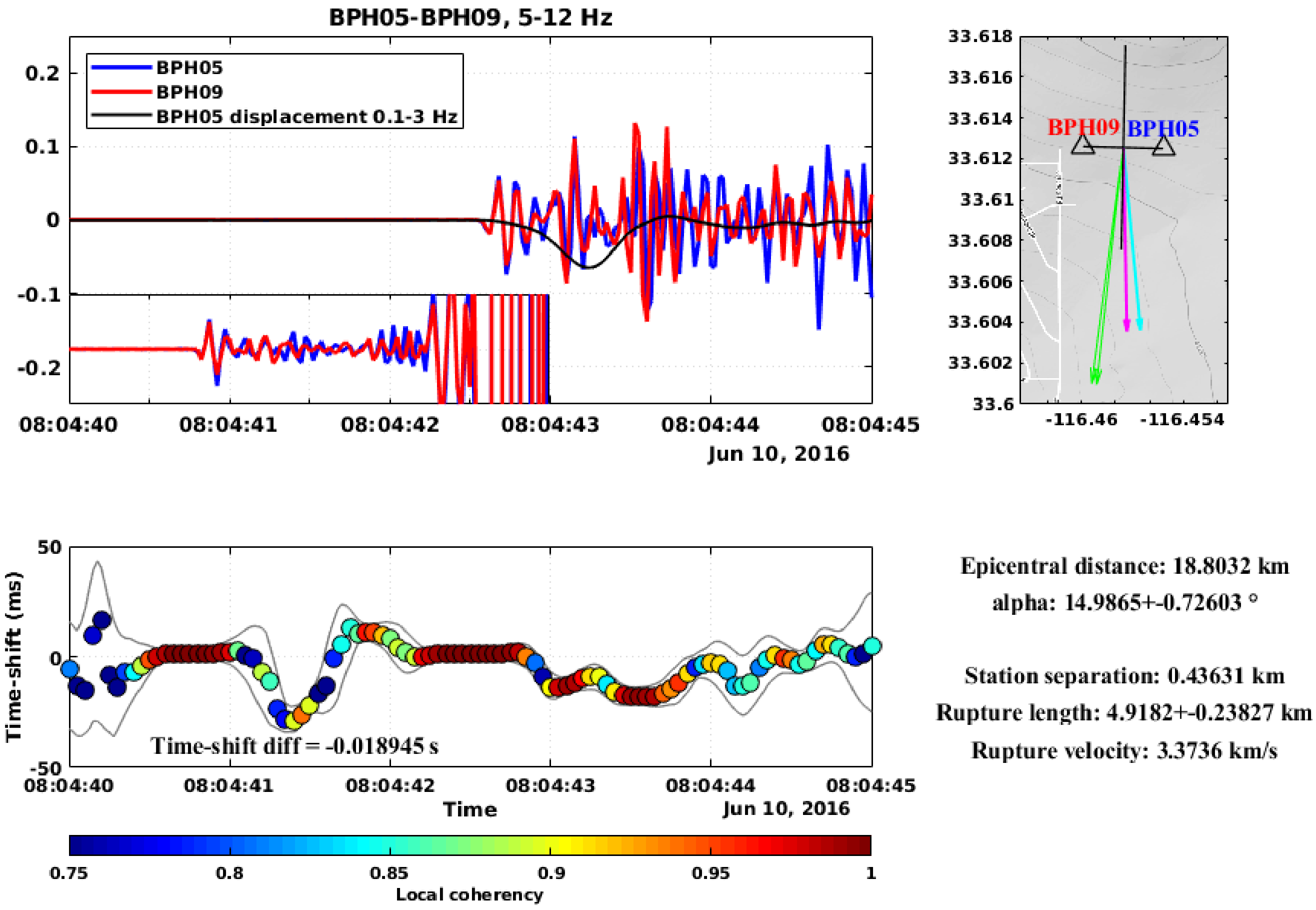

Figure S13. Stereometry analysis for station pair BPH04-BPH09.

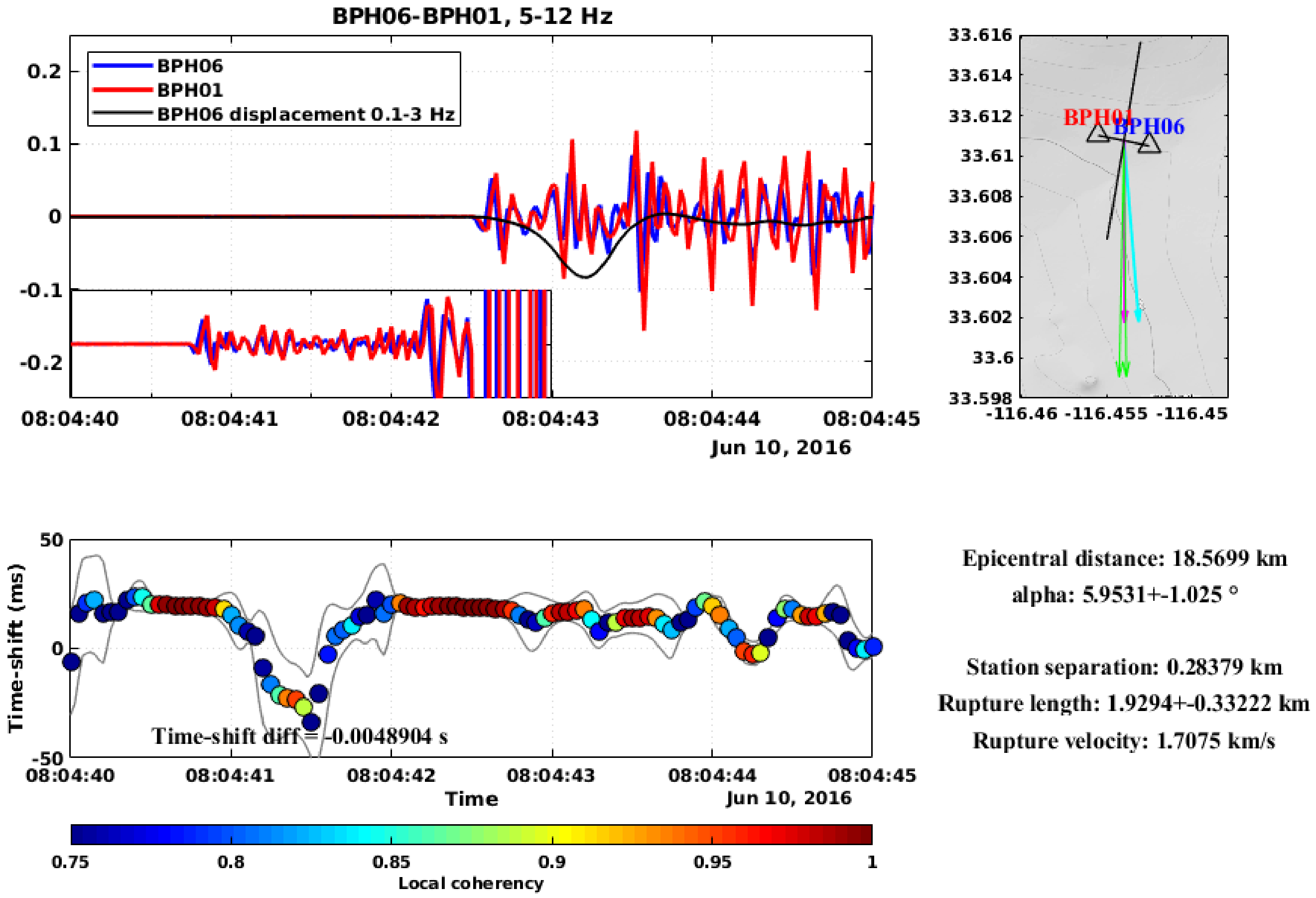

Figure S14. Stereometry analysis for station pair BPH06-BPH01.

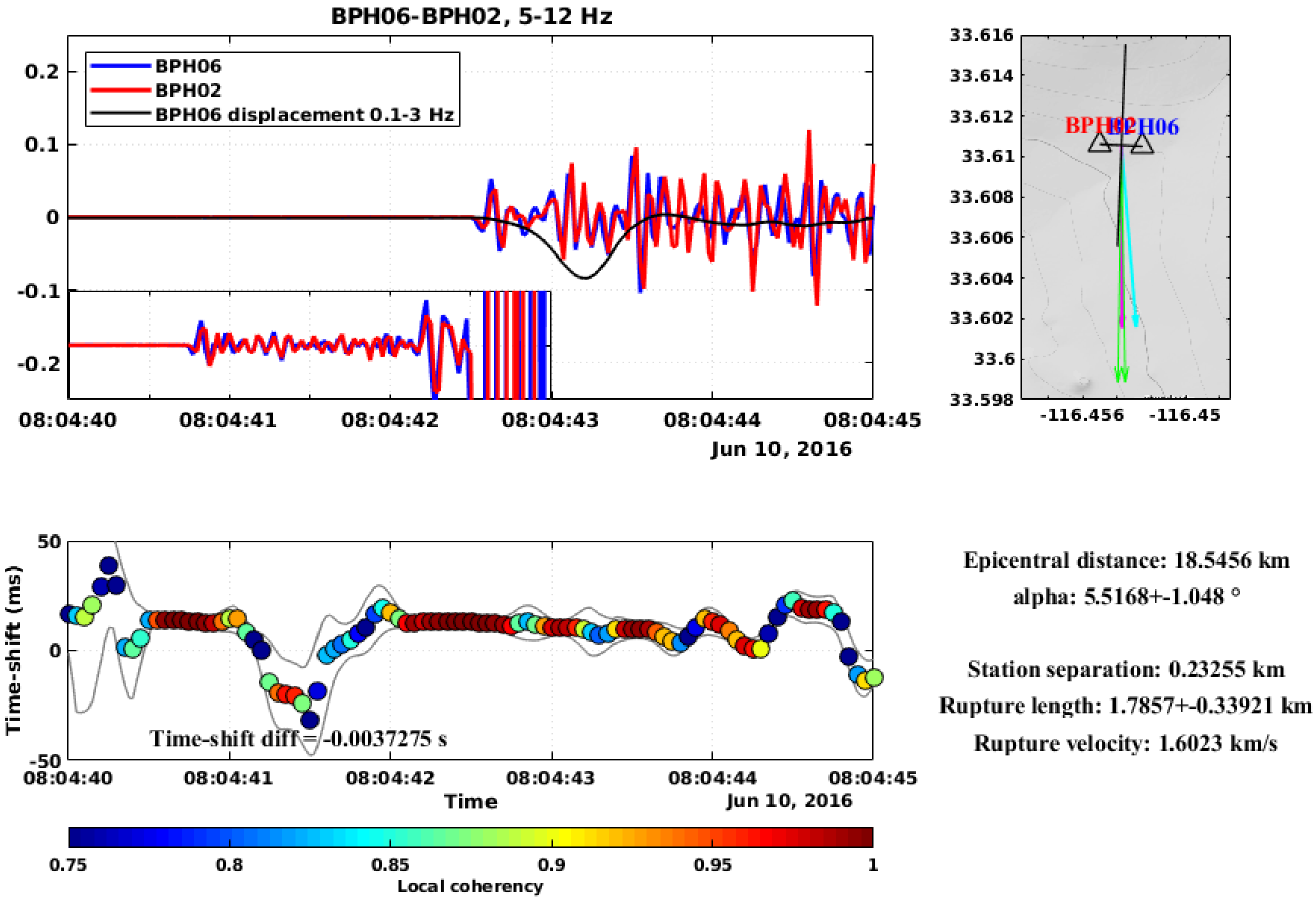

Figure S15. Stereometry analysis for station pair BPH06-BPH02.

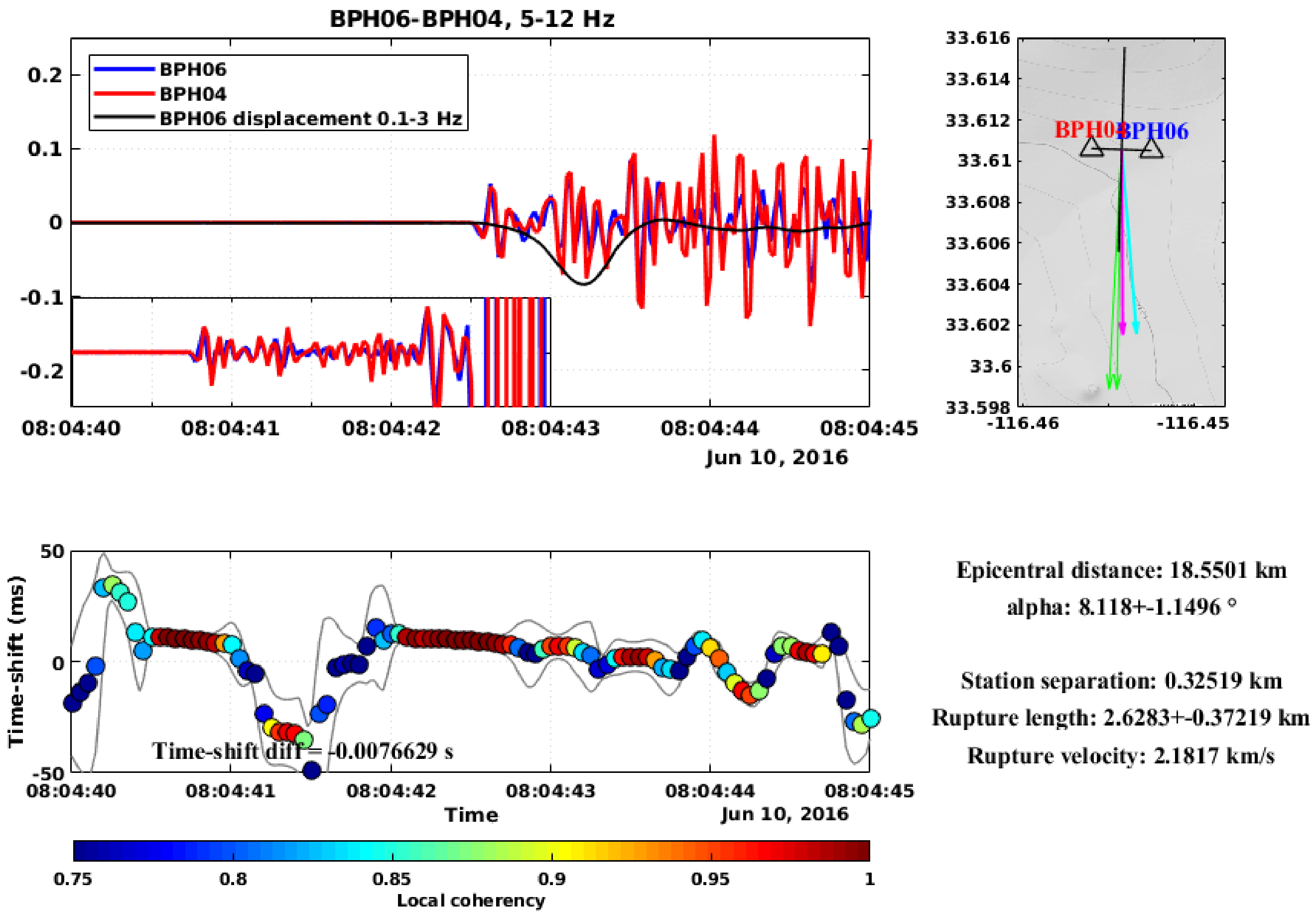

Figure S16. Stereometry analysis for station pair BPH06-BPH04.

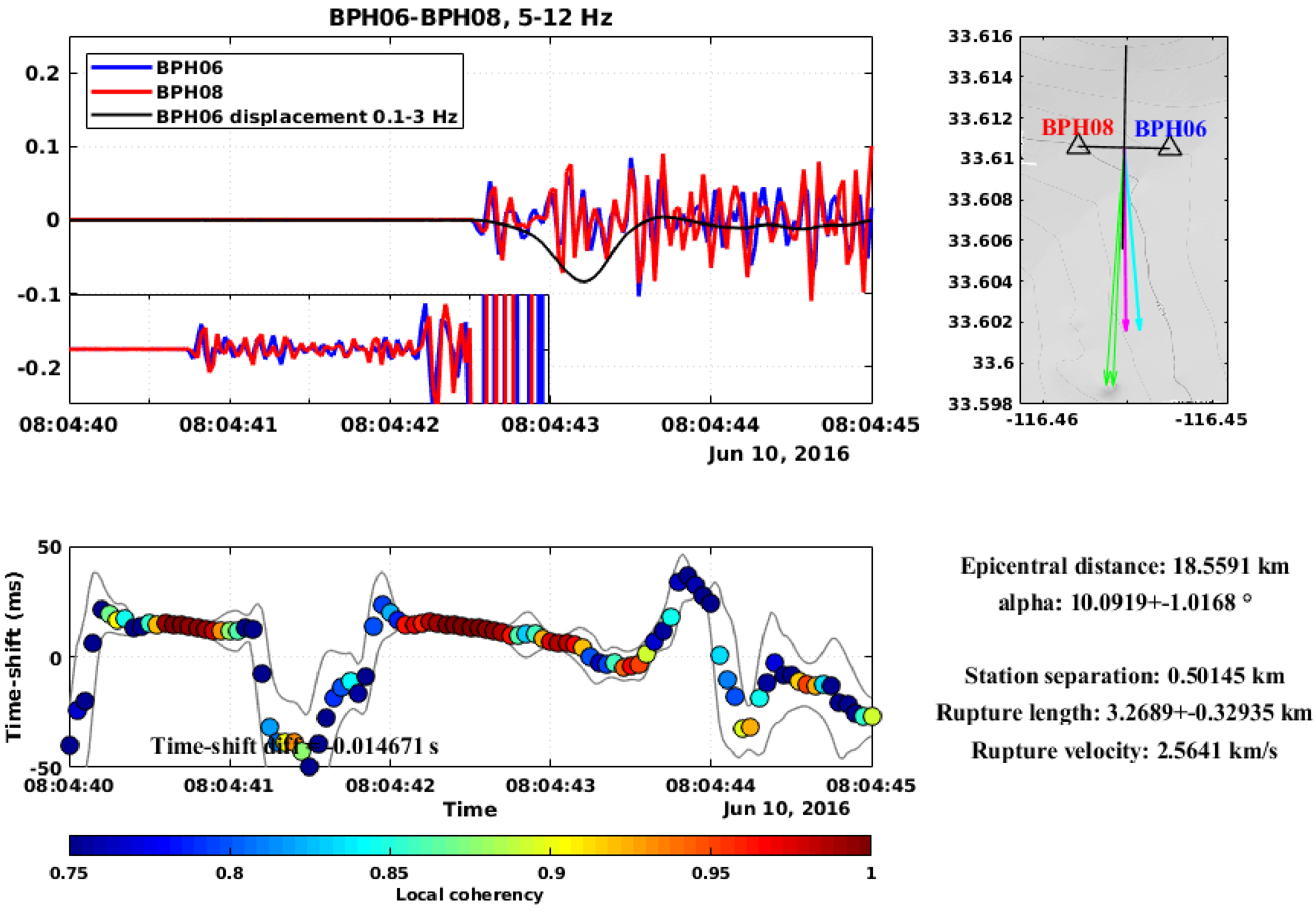

Figure S17. Stereometry analysis for station pair BPH06-BPH08.

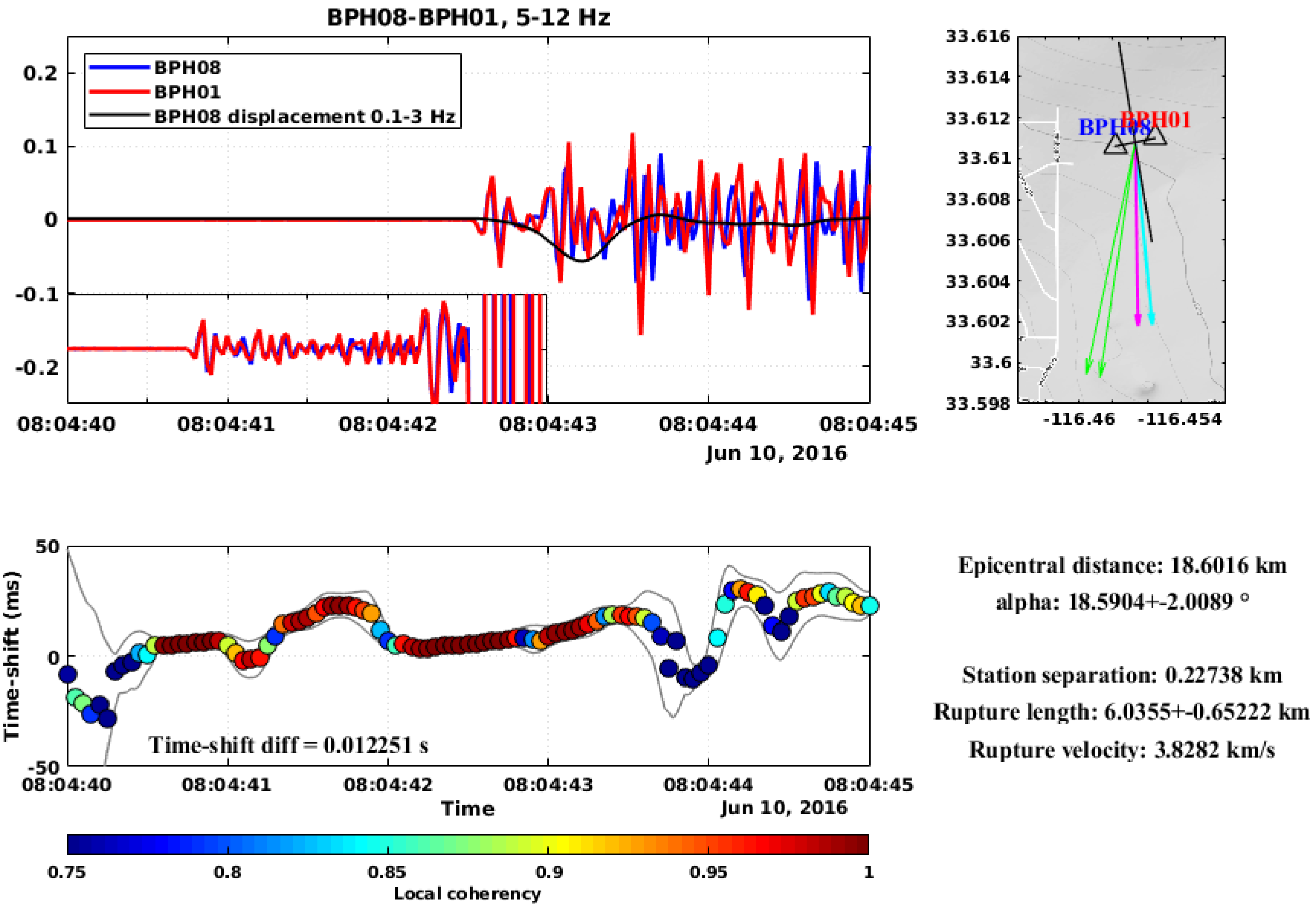

Figure S18. Stereometry analysis for station pair BPH08-BPH01.

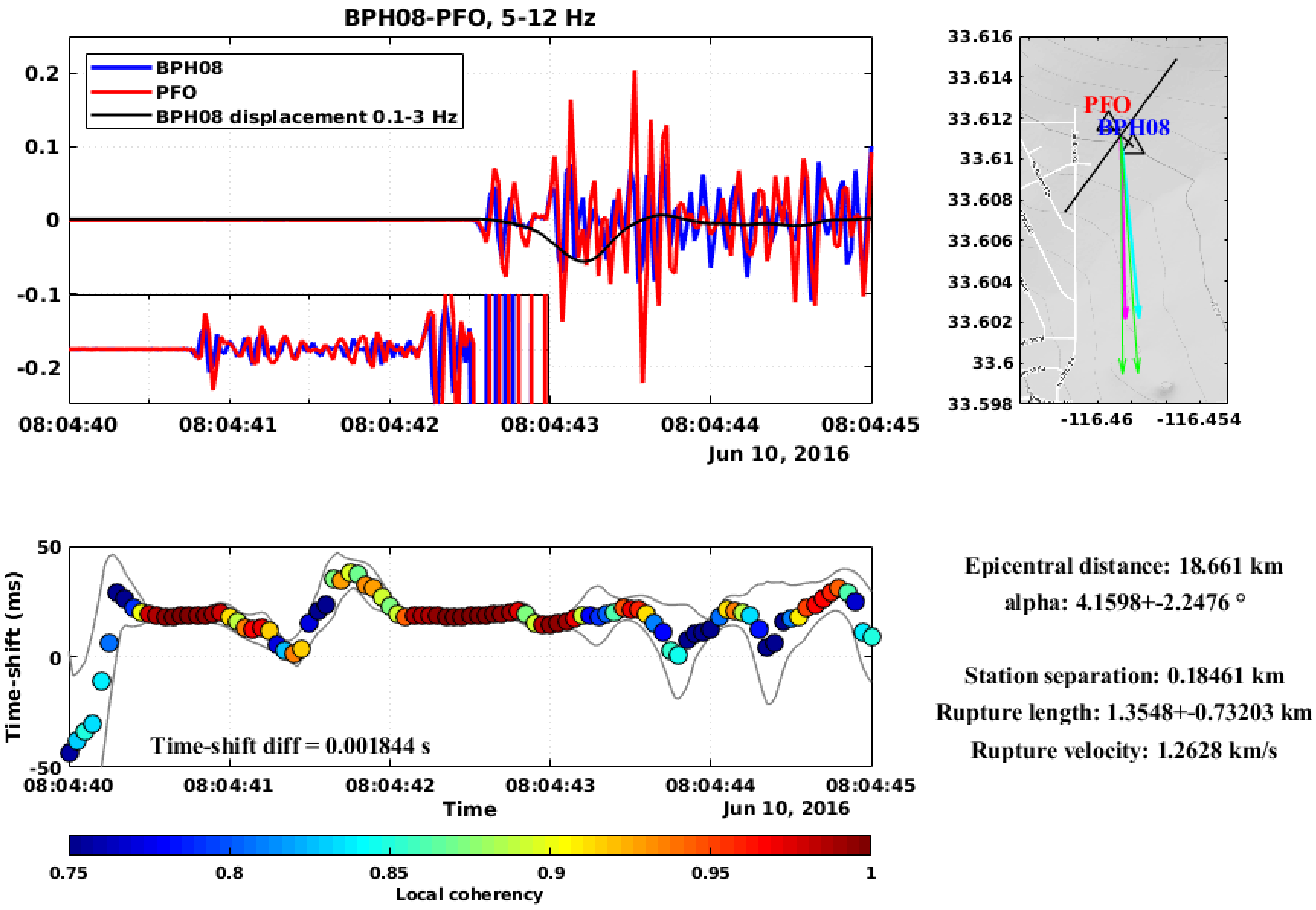

Figure S19. Stereometry analysis for station pair BPH08-PFO.

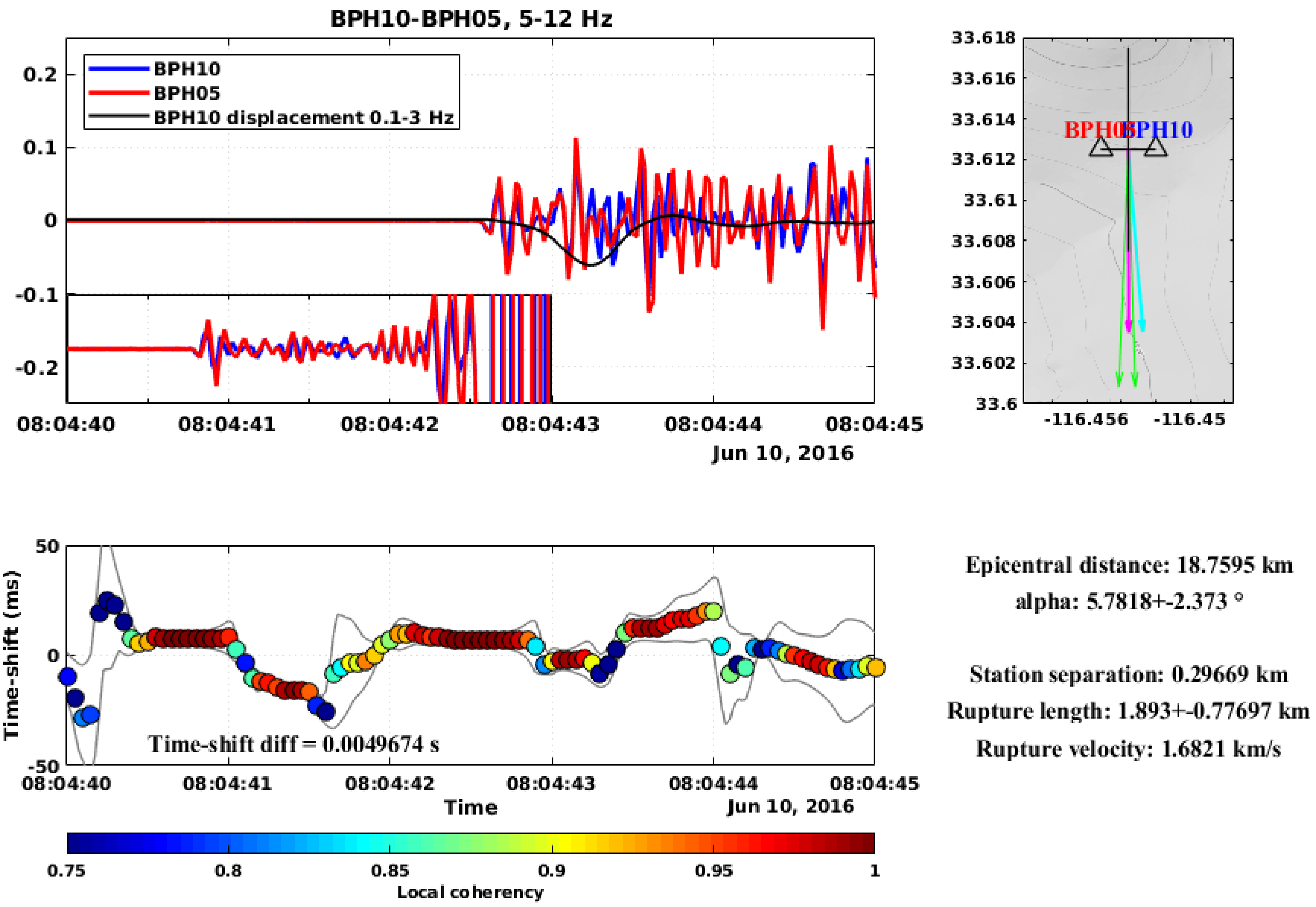

Figure S20. Stereometry analysis for station pair BPH10-BPH05.

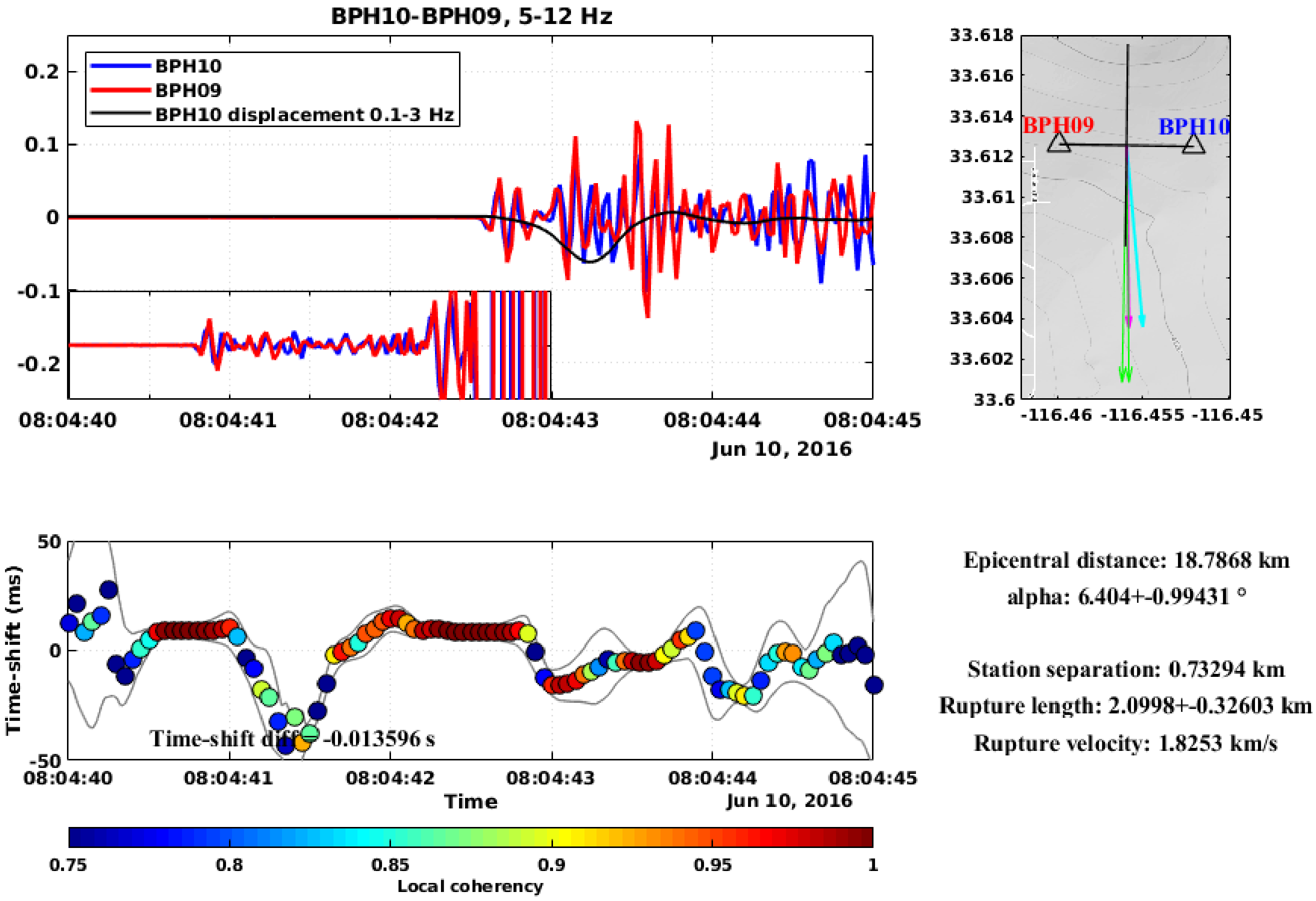

Figure S21. Stereometry analysis for station pair BPH10-BPH09.

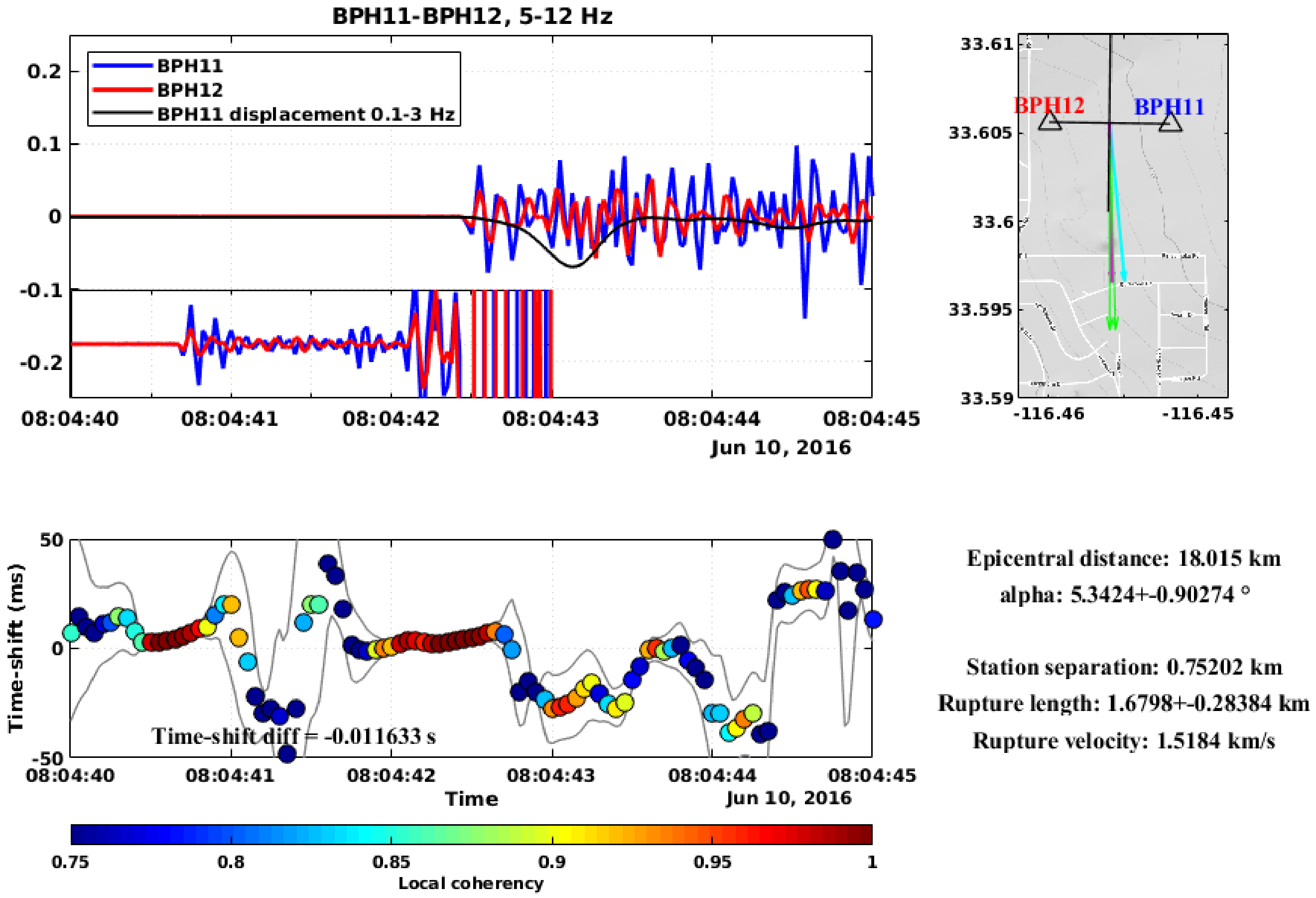

Figure S22. Stereometry analysis for station pair BPH11-BPH12.

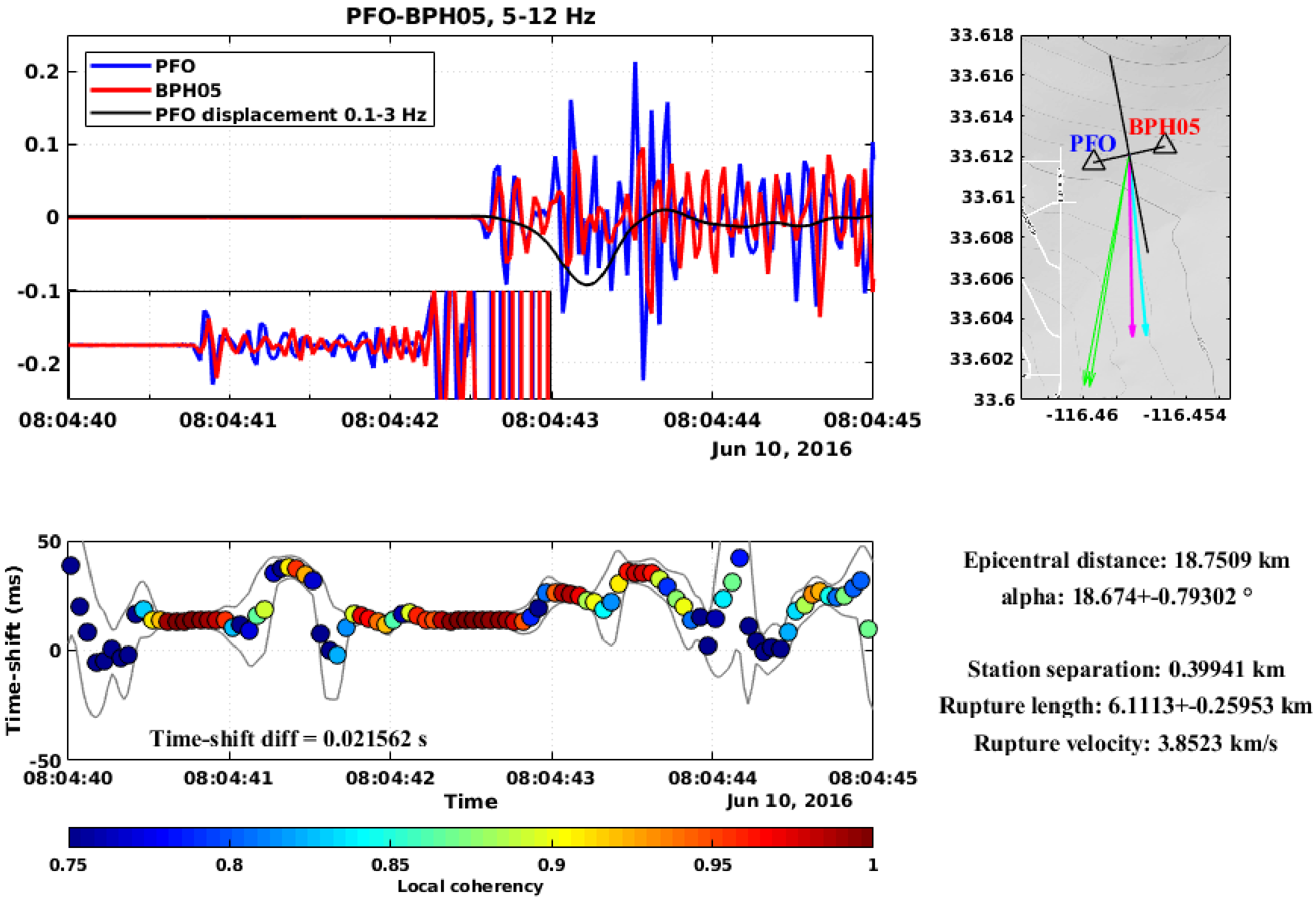

Figure S23. Stereometry analysis for station pair PFO-BPH05.

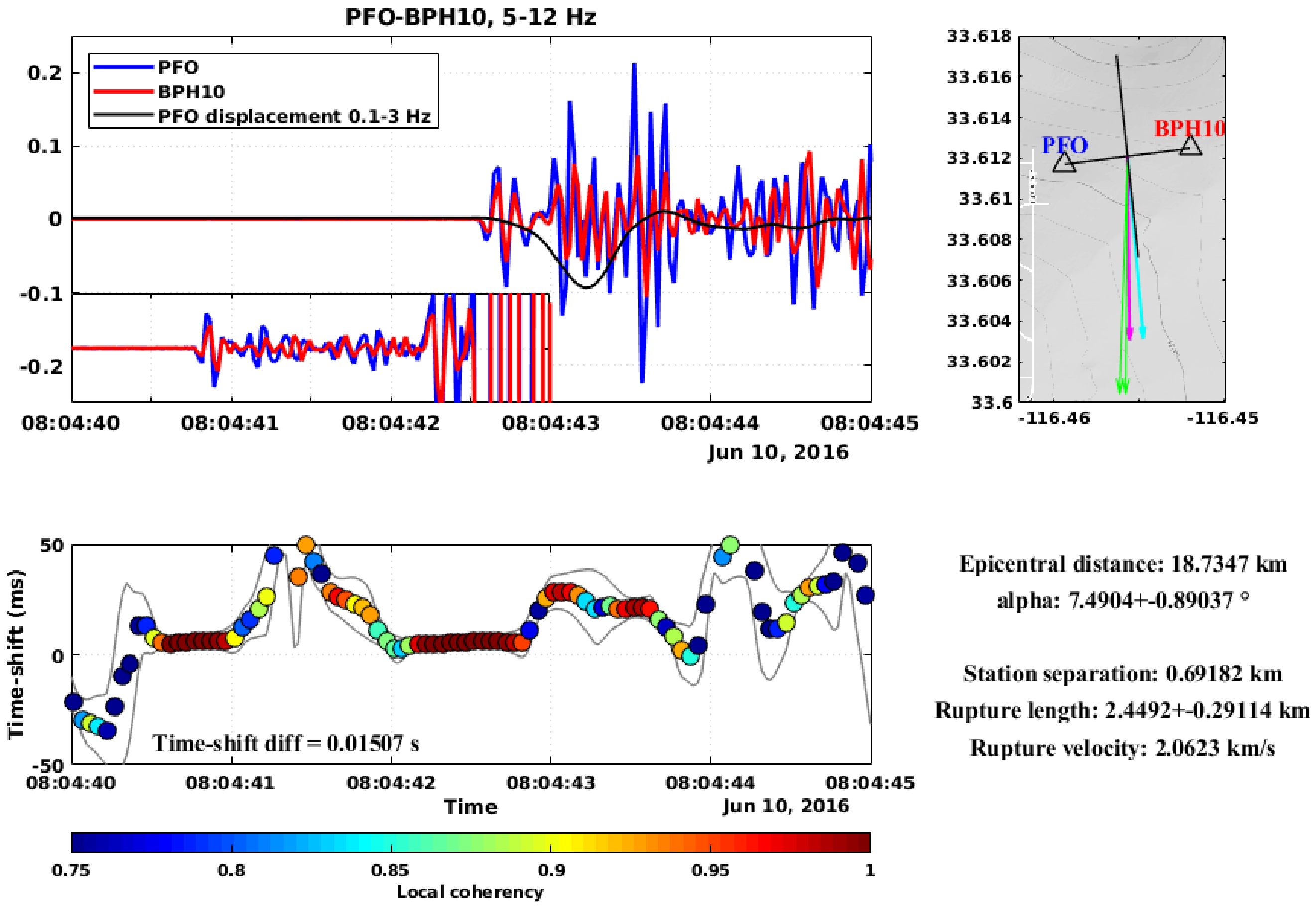

Figure S24. Stereometry analysis for station pair PFO-BPH10.

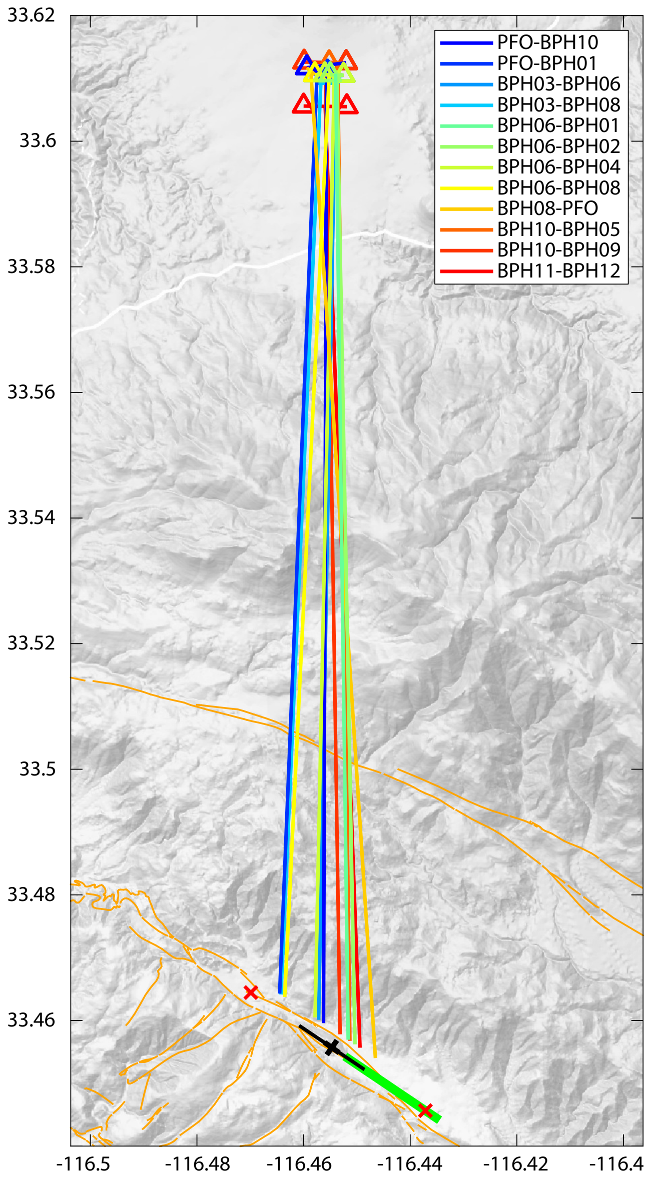

Figure S25. Estimation of the rupture length of the Borrego Springs earthquake using stereometry measurements. A colored line links the mid-point of each station pair to the coordinates of the end of the rupture. The green line is the rupture length estimated by Ross et al. (2017). The black cross is the median position for the end of the rupture, the black line is the uncertainty. The red crosses delineate the distance range in which the rupture length is searched for. The thin orange lines are the surface traces of Quaternary faults.

Table S1. Layered velocity model used for the synthetics

Depth (km)	Vp (km/s)	Vs (km/s)	Density (g/cm^3)
0	3.0	1.7	2.5
1	4.8	2.6	2.8
2	5.8	3.1	2.9
5	6.5	3.5	3.1
25	7.5	4.0	3.5

Table S2. List of Pinon Flat Observatory seismic stations used for the stereometry analysis with their coordinates.

Network	Station	Latitude	Longitude	Elevation
AZ	PFO	33.611698	-116.459396	1259
PY	BPH01	33.611000	-116.455498	1292
PY	BPH02	33.610600	-116.455002	1289
PY	BPH03	33.610199	-116.455498	1285
PY	BPH04	33.610600	-116.456001	1287
PY	BPH05	33.612499	-116.455200	1302
PY	BPH06	33.610500	-116.452499	1294
PY	BPH08	33.610600	-116.457901	1300
PY	BPH09	33.612598	-116.459900	1295
PY	BPH10	33.612499	-116.452003	1300
PY	BPH11	33.605499	-116.451897	1300
PY	BPH12	33.605598	-116.459999	1251
PY	BPH13	33.609200	-116.459999	1300
